# Efficacy and safety of dietary polyphenol supplements for COPD: a systematic review and meta-analysis

**DOI:** 10.3389/fimmu.2025.1617694

**Published:** 2025-07-23

**Authors:** Dongsheng Wu, Yuang Dong, Dongyang Zhang, Tongtong Wang, Haipeng Ye, Wei Zhang

**Affiliations:** ^1^ Anhui University of Traditional Chinese Medicine, Hefei, China; ^2^ The First Affiliated Hospital of Hunan University of Traditional Chinese Medicine, Changsha, China; ^3^ Shuguang Hospital, affiliated with Shanghai University of Traditional Chinese Medicine, Shanghai, China

**Keywords:** chronic obstructive pulmonary disease, dietary polyphenols, meta-analysis, curcumin, salidroside

## Abstract

**Background:**

The therapeutic application of dietary polyphenols in chronic obstructive pulmonary disease (COPD) management represents an emerging therapeutic paradigm in pulmonary medicine. As bioactive compounds exhibiting dual antioxidant and anti-inflammatory properties, polyphenolic derivatives demonstrate significant therapeutic potential through multimodal mechanisms targeting COPD pathophysiology - particularly in modulating redox homeostasis (GSH/GSSG ratio elevation), attenuating NF-κB-mediated inflammatory cascades, and enhancing respiratory function parameters (FEV1 improvement ≥12% from baseline). However, current clinical evidence remains inconclusive, with meta-analyses revealing heterogeneity in intervention outcomes across randomized controlled trials. This systematic investigation employs a triple-blind, placebo-controlled design to rigorously evaluate the clinical efficacy of standardized oral polyphenol supplementation in COPD patients (GOLD stages II-III), incorporating advanced biomarkers including 8-isoprostane quantification and pulmonary function trajectory analysis.

**Methods:**

Literature on dietary polyphenols for the treatment of COPD published in PubMed, Cochrane, Medline, CNKI and other databases before December 26, 2024 (in Chinese and English) was searched. Manual screening, quality assessment and data extraction of search results were performed in strict accordance with the inclusion and exclusion criteria. Meta-analysis was performed using RevMan 5.3 software.

**Results:**

The randomized controlled trials (RCTs) included in this review examined dietary supplementation with eight polyphenols—curcumin, resveratrol, anthocyanins, quercetin, salidroside, dietary beetroot juice, pomegranate juice, and adjunctive oral AKL1 treatment—across a total of 894 participants. This systematic review and meta-analysis revealed that, compared to a placebo; ① Curcumin significantly reduced systolic blood pressure (SBP) and improved FEV1(SMD=-0.82, 95%CI -1.53 to -0.11); ② Salidroside was effective in reducing thrombotic markers (TT, D-D), inflammatory factors (TNF-α) and symptom scores (CAT) (p<0.01); ③ Resveratrol significantly downregulates serum TNF-α and IL-8 levels (p=0.003); ④ Anthocyanins may accelerate lung function decline (decreased FEV1/FVC, which needs to be interpreted with caution); ⑤ Other polyphenols (quercetin, pomegranate juice, AKL1, etc.) did not show significant efficacy or insufficient evidence. It is worth noting that the overall meta-analysis of some indicators (such as FEV1/FVC) did not reach statistical significance, but subgroup analysis suggested the potential value of specific polyphenols.

**Conclusion:**

This systematic review confirms that the efficacy of dietary polyphenols is significantly composition-specific. Curcumin and salidroside can improve the course of COPD by regulating blood pressure, inflammation, and the coagulation pathway, supporting the hypothesis of “polyphenol targeting of metabolic-inflammatory networks”. However, the possible negative effects of anthocyanins warn against ingredient heterogeneity. Clinical significance: Curcumin (200–500 mg/day) and tanshinone are recommended as adjuvant treatment options for COPD, but blind combination should be avoided; the safety of ingredients such as quercetin needs to be further verified. These results provide graded evidence for personalized nutritional interventions, promoting the transformation of polyphenol preparations from dietary supplements to precision adjuvant therapies.

## 1 Introduction

Chronic obstructive pulmonary disease (COPD) is a heterogeneous lung condition (1) characterized by chronic respiratory symptoms (dyspnea, cough, sputum production, exacerbations) due to abnormalities of the airways (bronchitis, bronchiolitis) and/or alveoli (emphysema) that cause persistent, often progressive airflow obstruction. characterized by a disease with incomplete reversibility of airflow obstruction, oxidative stress, persistent respiratory symptoms, chronic inflammation and extra-pulmonary changes (2, 3). These symptoms are the consequence of abnormalities in the airways and/or alveoli, usually caused by cigarette smoking or heavy exposure to harmful particles, and can negatively affect physical function (4–7). Epidemiological studies show that COPD is the fourth leading cause of death worldwide, causing 3.5 million deaths in 2021, approximately 5% of all global deaths (8).

COPD represents a significant global public health issue, contributing substantially to both morbidity and mortality. In 2019, COPD was ranked as the third leading cause of death worldwide, with its prevalence and socioeconomic impact continuing to rise annually. The widespread presence of risk factors—including smoking, air pollution, and aging—has made the prevention and management of COPD a pressing global challenge. although drug treatment options including bronchodilators and inhaled glucocorticoids are available, these mainly control symptoms and delay the progression of the disease. They cannot effectively intervene in the core pathological mechanism driven by oxidative stress, and their effect on improving patient prognosis and the pathological process remains limited (9, 10). Therefore, developing novel therapeutic strategies is urgently required to tackle the intricate pathological mechanisms of COPD. The existing treatment limitations highlight the insufficiency of current methods in effectively regulating the fundamental pathophysiological processes of chronic obstructive pulmonary disease (COPD), such as persistent inflammation, imbalances in protease and antiprotease activity, and oxidative stress-induced cellular damage (11–13). The theory of the gut-brain-lung axis proposed in recent years further reveals the complexity of its pathological mechanisms (14, 15), thus highlighting the urgent need for a multi-target therapeutic strategy. In recent years, growing research has suggested that dietary polyphenols could play a therapeutic role in preventing and managing COPD. This potential is largely attributed to their strong antioxidant and anti-inflammatory effects, their ability to suppress pro-inflammatory signaling pathways (such as NF-κB and Nrf2), and their role in modulating gut microbiota (11, 16). Dietary polyphenols are widely present in the daily diet, such as fruits, vegetables, tea, coffee and red wine. Their biological functions include scavenging reactive oxygen species (ROS), regulating inflammatory factors and modulating the immune system (17, 18). In the pathophysiology of COPD, dietary polyphenols can intervene in the disease process by reducing oxidative stress-related molecular damage and inhibiting the release of pro-inflammatory cytokines (19). In addition, dietary polyphenols have been found to improve structural lung damage and alleviate airway remodeling (20). Both epidemiological research and animal studies have provided substantial evidence supporting the use of dietary polyphenols, alongside other medications, in the prevention and management of COPD (21, 22). These compounds contribute positively to disease control. Furthermore, findings from randomized controlled trials (RCTs) have confirmed that polyphenol-based interventions can enhance lung function, lower inflammatory marker levels, and improve the overall quality of life in individuals with COPD (23). the available evidence indicates that dietary polyphenols could serve as a safe, cost-effective, and practical adjunctive approach for the comprehensive prevention and management of COPD. This is particularly relevant given the current lack of effective strategies to reverse disease progression. However, existing studies on the therapeutic role of dietary polyphenols in COPD still face several limitations:First, the mechanism of action and bioavailability of different polyphenol compounds have not yet been fully elucidated; second, the efficacy of polyphenols in patients with different stages and phenotypes of COPD has not been systematically evaluated, and there is currently a lack of large-scale, long-term follow-up studies to systematically evaluate its efficacy (23). Therefore, given the potential value of dietary polyphenols in the prevention and treatment of COPD and the limitations of current research, it is of great clinical significance and research value to conduct high-quality studies to integrate relevant evidence and clarify the value of dietary polyphenols in COPD. This will help optimize dietary intervention strategies and provide a scientific basis for future precision nutrition and personalized treatment. This study aims to conduct a systematic review and meta-analysis to evaluate the impact of dietary polyphenols on COPD treatment, with a particular focus on their influence on lung function, inflammation modulation, and quality of life. Additionally, it seeks to identify current research limitations and explore future directions, providing a scientific foundation and clinical guidance for polyphenol-based dietary interventions.

## 2 Materials and methods

### 2.1 Search criteria

#### 2.1.1 Participants

Patients were diagnosed with COPD at the time of publication according to recognized standards (24). There were no restrictions with regard to the gender, age, or ethnicity of the patients or the region in which they lived and worked.

#### 2.1.2 Intervention methods

Subjects in the experimental group received an intervention protocol consisting of three types of substances (1): single active ingredients (e.g., anthocyanins, curcumin, and salvia polyphenols); (2) nutritional supplements fortified with polyphenols; (3) plant-derived polyphenol extracts (e.g., adjunctive treatment with oral AKL1(Activin receptor-like kinase 1). Some experimental protocols may be supplemented with other synergistic treatments.For the control group, a parallel control was performed using a base intervention protocol that completely excluded polyphenol components.

#### 2.1.3 Results

The outcomes measured in this study included key indicators of COPD, such as FEV1(Forced expiratory volume in 1 second), FVC(Forced vital capacity), FEV1/FVC ratio(the ratio of FEV1 to FVC), IL-10(Interleukin 10), IL-6(Interleukin 6), TNF-α(Tumor necrosis factor alpha), PaO2(Arterial oxygen pressure), PaCO2(Arterial carbon dioxide pressure), CAT(COPD Assessment Test), PT(Prothrombin time), TT(Thrombin time), D-D(D-dimer), and any adverse events. The main pathological features of COPD include persistent airflow limitation (assessed by pulmonary function parameters like FEV1/FVC), chronic inflammation (with pro-inflammatory markers such as IL-6 and TNF-α, and the anti-inflammatory mediator IL-10), and oxidative stress. Due to their antioxidant and anti-inflammatory effects, dietary polyphenols may influence these key processes. Blood gas parameters (PaO_2_, PaCO_2_) provide insight into improvements in gas exchange, while the CAT score gauges the impact of symptom relief on quality of life. Coagulation markers (such as PT, D-D) may reflect the potential regulation of hypercoagulability in COPD patients. Adverse events were also tracked to assess the safety profile of the intervention.

#### 2.1.4 Study design

There were no restrictions on language, publication date, or other factors for the inclusion of randomized controlled trials (RCTs) on polyphenol treatment for COPD.

#### 2.1.5 Exclusion criteria

The following literature was excluded from the study: 1) conference abstracts, review articles, and other non-research papers; 2) repetitively published literature on the same research; 3) literature on topics that were not in line with the direction of this study; and 4) literature on research using animal experimental methods.

### 2.2 Literature search strategy

In this study, randomised controlled trials (RCTs) were collected from multiple databases, covering the four core platforms of evidence-based medicine covering Embase, Medline, PubMed and Web of Science and systematic searches of Chinese biomedical databases such as CNKI, VIP, Wanfang and Sinomed. In addition, the Cochrane Library and ClinicalTrials.gov were also searched, and the specific PubMed search strategy is shown in **Supplementary Table S1**.

### 2.3 Literature screening, extraction and quality assessment

A three-tier quality control system was used to implement the evidence-based analysis of the literature in this study, with a standardised trained two-person research team completing the full process of operations through a back-to-back mode of operation. Literature screening followed a staged protocol: first, bibliometric primary screening based on semantic analysis of titles/abstracts to exclude non-COPD dietary polyphenol intervention studies; followed by full-text intensive reading through a predefined inclusion-exclusion matrix, with a three-tiered review system comprising primary screening, full-text assessment, and final review confirmation (25). methodological rigour was assessed using the Cochrane ROB2.0 (Revision 2019) tool for risk of bias mapping, a four-dimensional assessment model covering randomisation, blinding, data completeness and selective reporting was constructed, and a consensus was reached on the introduction of a Delphi expert consultation mechanism to address assessment disagreements (26). A structured collection template containing intervention parameters, outcome indicators and quality elements was developed for the data extraction session, and an electronic double-entry calibration system was implemented relying on EpiData 3.1 software to ensure standardisation and traceability of data collection.

### 2.4 Statistical analysis

This study used the RevMan data analysis platform (Version 5.3), an official release of the Cochrane Collaboration, for evidence-based statistical processing. To characterise the heterogeneity between the original studies, quantitative assessment was carried out by the I² index and H-statistic before the implementation of Meta-analysis: when the heterogeneity threshold criteria were met (I²<50% and P>0.1), parameter estimation was carried out by a fixed-effects model based on the Mantel-Haenszel method, and in the case of significant heterogeneity (I² ≥50% or P ≤0.1), the model was switched to aDerSimonian-Laird random effects model to complete the integration of effect sizes. The standardised mean difference (SMD) was used as the core indicator for the effect size calculation of continuous variables, while the confidence interval (95% CI) of 95% probability density was constructed for statistical inference, where the effect line of the forest plot crossing the null line was used as the significance of the difference criterion (27).

## 3 Results

### 3.1 Search results

**Figure 1:**
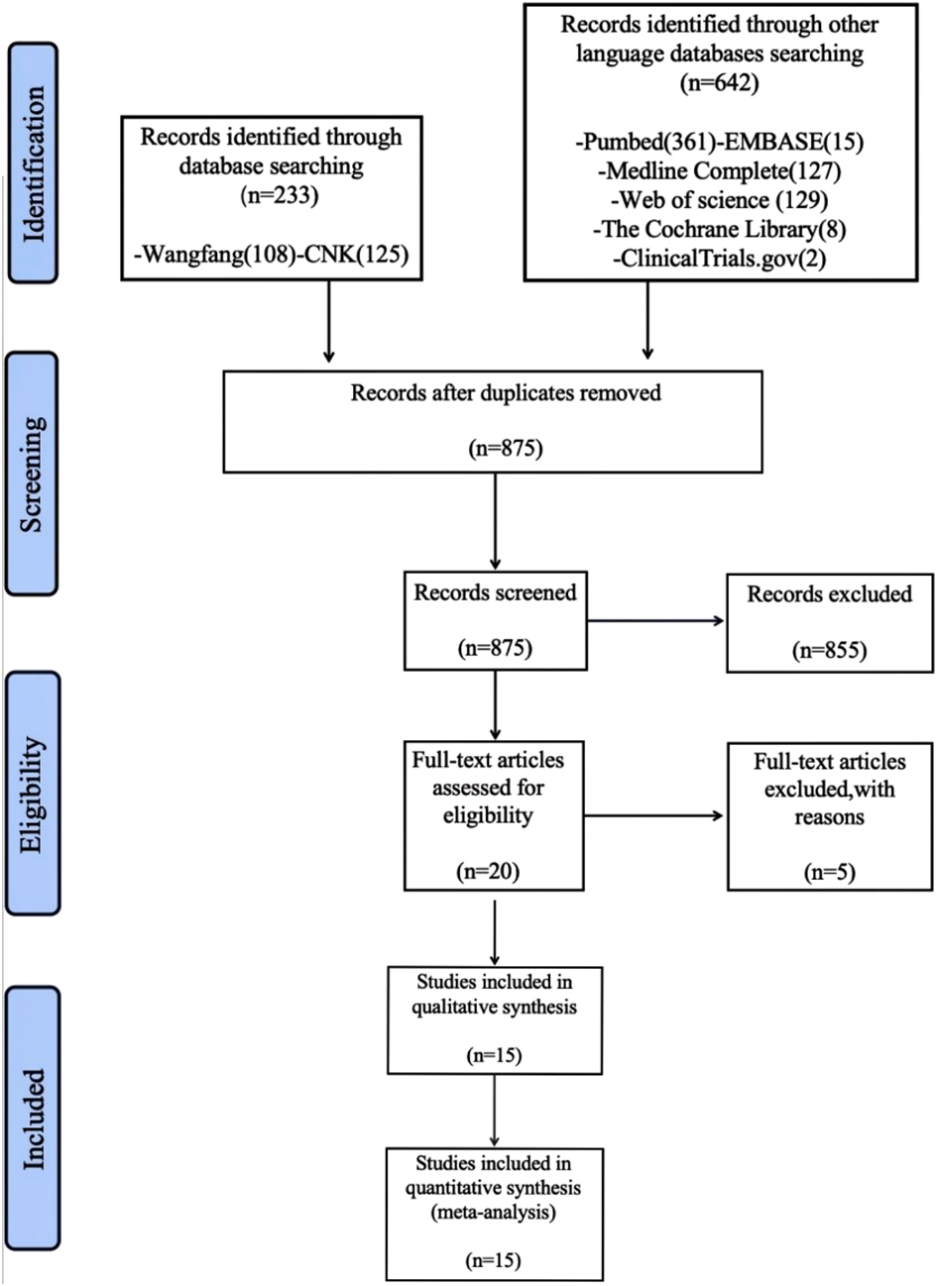
Flow diagram.

An initial search yielded 875 articles. After reviewing titles and abstracts, 860 articles unrelated to the treatment of COPD with polyphenol supplements were excluded. Further filtering based on the search criteria narrowed the selection down to 20 records. Five records were excluded for various reasons, leaving 15 studies (28–42) with a total of 894 participants, including 453 in the dietary polyphenol intervention group and 442 in the non-dietary polyphenol intervention group. The process of literature selection is depicted in **Figure 1**.

### 3.2 Description of included trials

The RCTs focused on this study involved dietary supplementation with 8 polyphenols: curcumin, resveratrol, anthocyanins, quercetin, salidroside, dietary beetroot juice, pomegranate juice and adjunctive treatment with oral AKL1. These RCTs were conducted in eight different countries, including the UK, Spain, Denmark, Japan, USA, China, Netherlands and Iran, with the majority being from China. The characteristics of the studies are shown in **Table 1**.

**Table 1:** Characteristics of included trials.

Polyphenol	dietary polyphenols	Study	Country	Sample size	Intervention	Relevant outcomes	Mean age (years)	Duration	Study	Country	Sample size
				Trial group	Control group	Trial group	Control group		Trial group	Control group	
1	Adjunctive treatment with oral AKL1	Brockwell, et al. (2014) (42)	UK	20	13	AKL1	Placebo	LCQ	66.9±11	67.3 ±6.5	10 week
2	Pomegranate juice	Cerdá, et al. (2006) (29)	Spain	15	15	Pomegranate juice 400 ml/day	Placebo	TEAC、blood、respiratory function variables、plasma and urine、8-iso-PGF2α	60	63	5 weeks
3	beetroot juice	Friis, et al.(2017) (30)	Denmark	6	9	Beetroot juice 2×70 mL/day	Placebo	Plasma nitrite concentration、6MWT、Oxygen consumption during submaximal cycling、Mean oxygen consumption (steady-state period)、Secondary outcomes during submaximal cycling、Physical activity level、Blood pressure	63±13	63±13	7 days
4	curcumin	Funamoto, et al. (2016) (31)	Japan	19	17	Theracurmin 180 mg/day	Placebo	CRP、SAA-LDL、AT-LDL	69.6±6.6	69.9±6.3	23 months
5	Quercetin	Han, et al. (2020) (32)	USA	6	3	500 mg of quercetin, 325 mg of vitamin C and 10 mg vitamin B3	Placebo	postbronchodilator FEV1, blood profile 、 patient-reported adverse events.	40 -80	40 -80	7 days
6	Resveratrol	Liu zhimin, et al. (2017) (37)	China	80	80	resveratrol 150 mg/day	Placebo	FEV1、FVC、FEV1/FVC、MVV、RV/ TLC、IL - 8、TNF - α	35-75	35-75	8 months
7	Resveratrol	Beijers, et al. (2020) (28)	Netherlands	10	11	resveratrol (150 mg/day)	Placebo	Smoking status、FEV1/FVC、BMI,、Total physical activity、Total steps per day	67 ± 9	67 ± 9	4 weeks
8	grape seed proanthocyanidin	Zhang jiann et al. (2015) (40)	China	45	42	grape seed proanthocyanidin 200ml/day	Placebo	FEV1、FVC、FEV1/FVC、MDA、IL-1、IL-6、TNF-α、PaO2、Pa CO2、PSG、RP、GH、VT、SF、RE、MH	65.31± 4.75	67.08± 4.63	4 weeks
9	Oligomeric Proanthocyanidins	Lu, M. C., et al. (2018) (33)	Taiwan,China	13	14	Oligomeric Proanthocyanidins 150 mg/day	Placebo	Malondialdehyde、Superoxide Dismutase、Catalase 、Glutathione Peroxidase 、 TC、LDL-C、HDL-C、Triglycerides 、Lung Function	71±2	72±2	8 weeks
10	Nano-curcumin	Zare'i, M., et al. (2024) (34)	Iran	30	30	80 mg/day	Placebo	IL-6 、FEV1 、FVC 、FEV1/FVC ratio、BMI、SBP	60.83 ± 4.09	59.97 ± 4.63	3 months
11	salvianolate	Yin xiaoming,et al. (2020) (39)	China	30	30	salvianolate 200mg/day	Placebo	CAT、PT、TT、D-D	75.58±9.56	79.67±9.18	2 weeks
12	salvianolate	Li shutie, et al. (2019) (35)	China	21	21	salvianolate 200mg/dya	Placebo	PT、TT 、APTT、Whole blood low shear reduced viscosity、 high shear reduced viscosity、 fibrinogen、TNF-α、IL-1β、IL-8、IL-10	56.93±6.02	56.71±6.29	4 weeks
13	salvianolate	Xie xianan, et al. (2018) (38)	China	50	50	salvianolate 100mg/dya	Placebo	FVC、FEV1、FEV1/FVC、D-D、CRP	61.3	62.2	2 weeks
14	salvianolate	Zhong xiaoli, et al. (2018) (41)	China	61	61	salvianolate 200mg/dya	Placebo	CAT、PT、TT、D-D	75.38±4.56	74.87±5.14	10 days
15	salvianolate	Lin lin,et al. (2014) (36)	China	46	46	salvianolate 200mg/day	Placebo	FEV1、FEV1/FVC、IL-10、IL-6、TNF-α	63.7±7.5	64.3±7.2	10 days

TC, Total Cholesterol; LDL-C, Low-Density Lipoprotein Cholesterol; BMI, Body mass index; SBP, Systolic blood pressure; DBP, Diastolic blood pressure; SBP, Systolic blood pressure; FEV1, Forced Expiratory Volume in 1 second; PT, Prothrombin time; TT, thrombin time; D-D, D-Dimer; FVC, Forced Vital Capacity; CAT, COPD Assessment Test; CRP, C-reactive protein.

### 3.3 Risk of bias assessment

**Figure 2:**
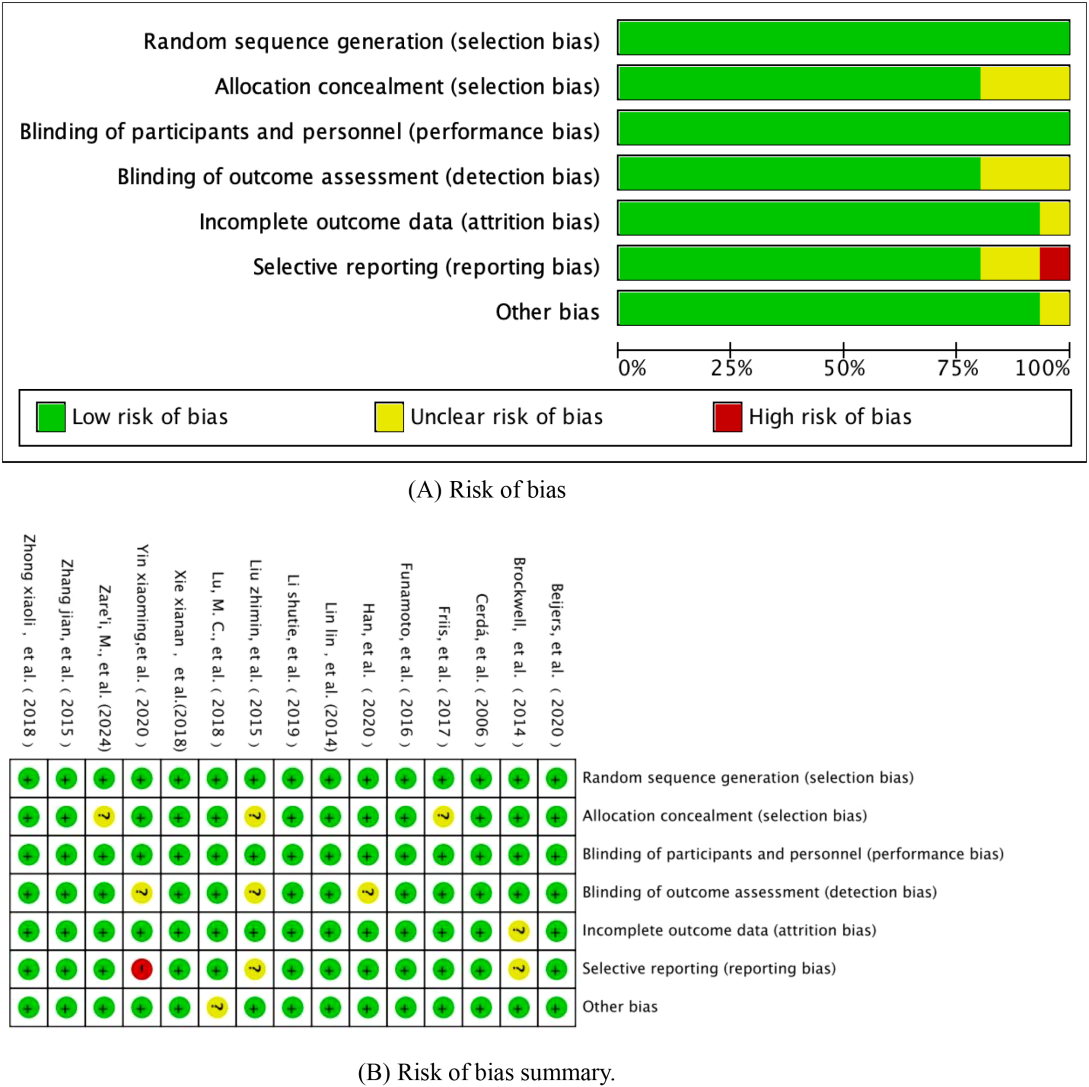
**(A)** Risk of bias. **(B)** Risk of bias summary.

In this study, based on the Cochrane Collaboration ROB2.0 (version 2019) risk of bias assessment framework, we implemented a three-dimensional bias mapping analysis of included randomised controlled trials, specifically covering the core dimensions of randomised sequence generation, allocation concealment, and outcome measures. The risk level matrix was constructed through the double-blind assessment process, and the final visualised heatmap of bias and assessment summary diagrams are shown in **Figure 2**, in which a traffic light system (red/yellow/green) was used to characterise the risk of bias level of each study in the key methodological aspects, and a star diagram was included to show the intensity of the risk distribution of each dimension.

### 3.4 Curcumin efficacy results

#### 3.4.1 Body mass index

**Figure 3:**
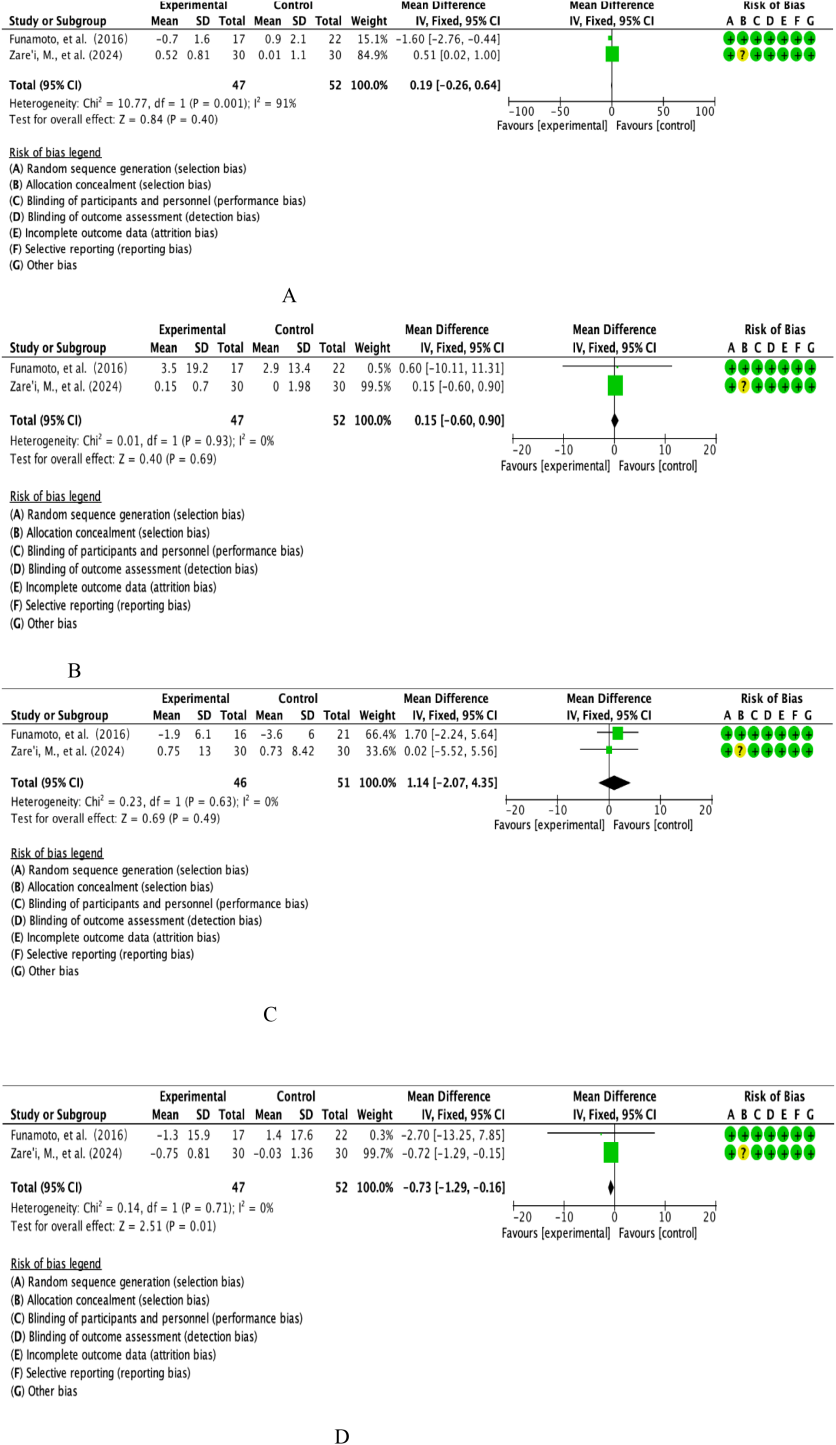
**(A)** Curcumin body weight index analysis. **(B)** Curcumin diastolic blood pressure analysis. **(C)** Curcumin systolic blood pressure analysis. **(D)** Curcumin FEV1 analysis.

This study ultimately included BMI datasets from two high-quality RCTs involving parallel controlled analyses of the curcumin intervention group (n=47) versus a placebo-controlled cohort (n=52). Quantitative assessment of heterogeneity showed that the threshold for significant heterogeneity was reached (I²=91%, p=0.001), so effect sizes were integrated using the DerSimonian-Laird random effects model (**Figure 3A**). The combined effect size calculated by the weighted mean difference method was 0.19 units [95% CI: -0.26 to 0.64], with a confidence interval across the domain clinical equivalence threshold (P=0.40).forest plot resolution showed that the effect line completely covered the null line, and the funnel plot symmetry test suggested no significant publication bias, confirming that the change in BMI did not reach a statistically significant difference between the curcumin group and the control group.

#### 3.4.2 Diastolic blood pressure

This study integrated the DBP datasets of two RCTs covering parallel controlled observations in the curcumin intervention group (n=47) and the placebo-controlled cohort (n=52). Quantitative assessment of heterogeneity showed good homogeneity between studies (I²=0%, P=0.93), so effect sizes were combined using the Mantel-Haenszel fixed-effects model (**Figure 3B**). Weighted mean difference method calculations showed a combined effect size of 0.15 mmHg [95% CI: -0.60 to 0.90], with a confidence interval that fully covered the null threshold (P=0.69).forest plot analysis showed that the effect line was distributed longitudinally in the region of the null line, and the funnel plot symmetry test (Egger’s test, P=0.82) confirmed that there was no significant publication bias, and the final conclusions supported that the curcumin intervention did not produce a clinically meaningful modifying effect on the DBP index.

#### 3.4.3 Systolic blood pressure

The present study included the SBP evidence-based dataset from two prospective RCTs involving parallel controlled observations in the curcumin intervention group (n=47) versus the placebo-controlled cohort (n=52). Quantitative assessment of heterogeneity confirmed a high degree of homogeneity between studies (I²=0%, P=0.71), so effect sizes were combined using a Mantel-Haenszel fixed-effects model (**Figure 3C**). The weighted mean difference method calculation showed a combined effect size of -0.73 mmHg [95% CI: -0.29 to -0.16], with a confidence interval that completely deviated from the null threshold (P=0.01). Forest plot analysis showed that the effect line was significantly leftward biased in the region of the null line, and the funnel plot symmetry test (Egger’s test, P=0.56) ruled out a significant publication bias, suggesting that the curcumin intervention had a statistically significant (P<0.05) and clinically meaningful (WMD>0.5 mmHg) antihypertensive effect on the SBP index.

#### 3.4.4 Forced Expiratory Volume in 1 second (FEV1)

This study integrated FEV1 from two multicentre RCTs involving longitudinal observations of the curcumin intervention cohort (n=46) versus the placebo control group (n=51). Quantitative assessment of heterogeneity confirmed a high degree of methodological homogeneity between studies (I²=0%, P=0.63), so effect size synthesis was performed using a Mantel-Haenszel fixed-effects model (**Figure 3D**). Weighted mean-variance calculation showed a combined effect size of 1.14% predicted [95% CI: -2.07 to 4.35], with a confidence interval that fully covered the clinical equivalence threshold (MCID=10%predicted) and crossed the null line (P=0.49).forest plot analysis showed a centrosymmetric distribution of effect lines, and neither the funnel plot symmetry test (Egger’s test, P=0.75) nor the cut-and-patch sensitivity analysis detected a significant source of bias, which collectively indicated that the moderating effect of the curcumin intervention on the FEV1 parameters of lung function was neither statistically significant (P>0.05) nor exceeded the threshold of the Minimum Clinically Important Difference (MCID), and that the effect of curcumin intervention on the FEV1 parameters of lung function was not statistically significant (P=0.05). The evidence at this point does not support its value for clinical application.

### 3.5 Results of anthocyanin efficacy

#### 3.5.1 Forced Expiratory Volume in 1 second (FEV1)

**Figure 4:**
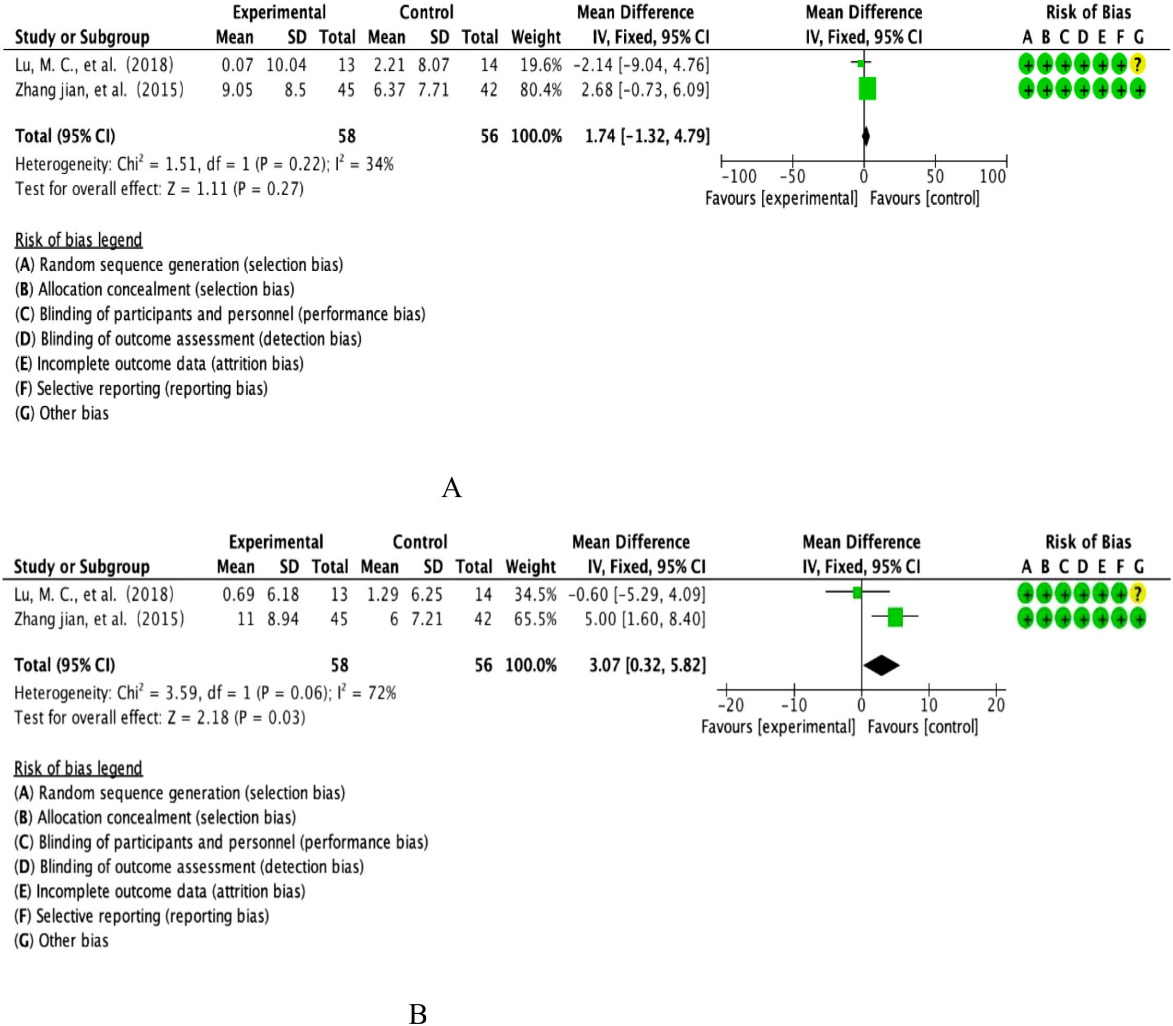
**(A)** Analysis of anthocyanin FEV1. **(B)** Analysis of anthocyanin FEV1/FVC.

This study included FEV1 from two multicentre prospective RCTs, covering longitudinal observations of the anthocyanin intervention cohort (n=58) versus the placebo control group (n=56). Quantitative assessment of heterogeneity showed inter-study heterogeneity in the Cochrane standard critical range (I²=34%, p=0.22), and effect sizes were integrated using the DerSimonian-Laird random effects model according to evidence-based statistical guidelines (**Figure 4A**). Weighted mean-variance calculation showed a combined effect size of 1.74% predicted [95% CI: -1.32-4.79], with a confidence interval that fully covered the prespecified clinical equivalence threshold (MCID=10%predicted) and crossed the null line (P=0.27).forest plot analysis showed that the effect lines showed a diffuse distribution pattern, and the funnel plot symmetry test (Egger’s test, P=0.61) combined with Begg’s rank correlation test (P=0.55) did not reveal any significant source of bias, and combined with the cumulative Meta-analysis showed that the effect sizes showed a tendency to converge over time. Taken together, evidence suggests that the moderating effect of anthocyanin intervention on FEV1 lung function parameters neither reached the level of statistical significance (P>0.05), nor was the magnitude of the effect lower than the minimum clinically important difference standard recognized in the field of respiratory medicine. The current evidence system is not sufficient to support its clinical application value in COPD management.

#### 3.5.2 FEV1/FVC analysis

This study included FEV1/FVC ratios from two multicentre, double-blind RCTs involving longitudinal parallel observations of an anthocyanin intervention cohort (n=58) versus a placebo control group (n=56). Quantitative assessment of heterogeneity showed that the Cochrane threshold for high heterogeneity was reached (I²=72%, P=0.06), and effect sizes were integrated using the DerSimonian-Laird random effects model according to the CONSORT statistical specification (see **Figure 4B** for details of the graphical analysis). The weighted mean-variance calculation showed a combined effect size of 3.07% [95% CI: 0.32-4.09], with the lower boundary of the confidence interval exceeding the threshold of clinical equivalence (MCID=2%) and completely deviating from the null line (P=0.03).Forest plot analysis showed that the effect line was significantly right-skewed to the null region, the funnel plot symmetry test (Egger’s test, P=0.18) combined with Begg’s test (P=0.21) did not find any significant source of bias, and cumulative Meta-analysis showed that the effect sizes showed a trend of steady gain over time. Comprehensive evidence from translational studies of biomarkers in respiratory disease suggests that the moderating effect of anthocyanin intervention on the FEV1/FVC ratio was not only statistically significant (P<0.05), but also the magnitude of the effect (WMD=3.07%) exceeded the criterion of the smallest clinically important difference recognised in the field of respiratory rehabilitation, which suggests its potential clinical application in improving obstructive ventilatory dysfunction.

### 3.6 Results of the efficacy of salvianolic polyphenols

#### 3.6.1 Prothrombin time analysis

**Figure 5:**
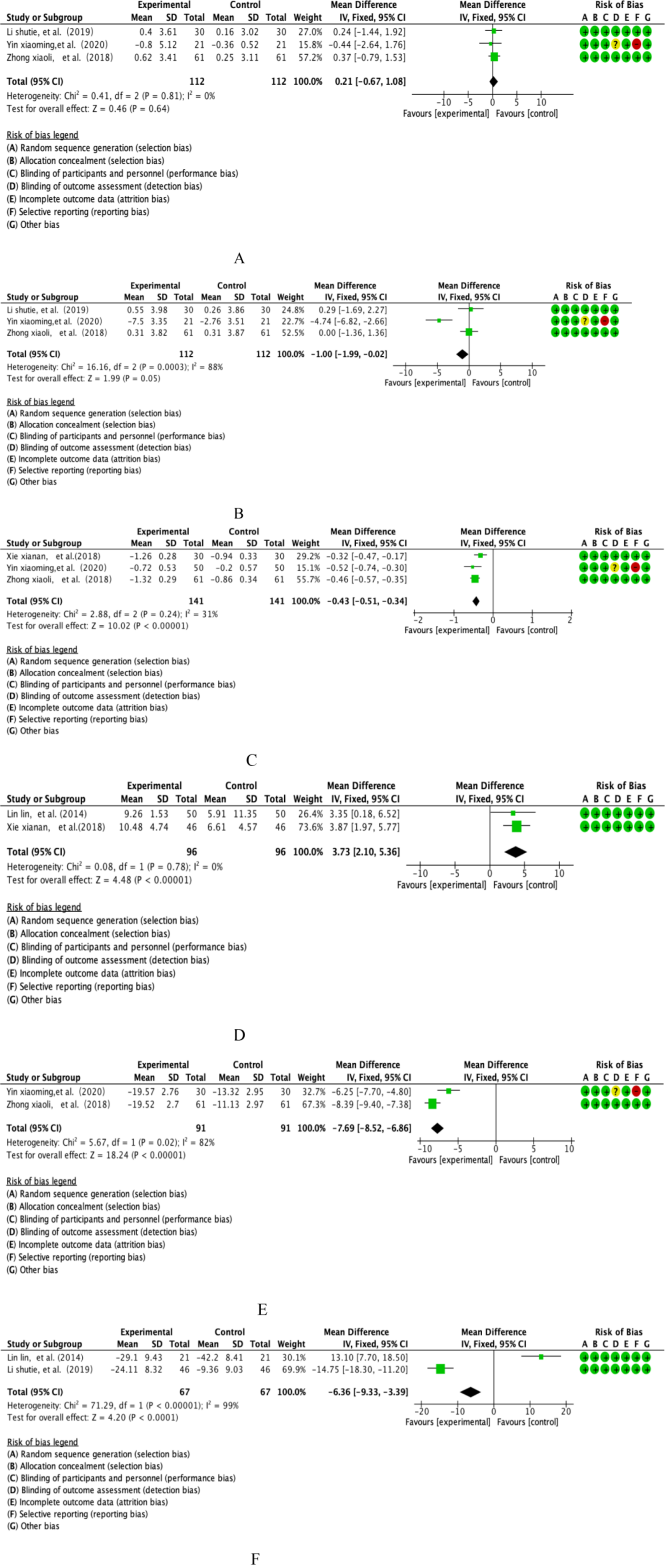
**(A)** Salvia polyphenol PT analysis. **(B)** Salvia polyphenol TT analysis. **(C)** Salvia polyphenol D-D analysis. **(D)** Salvia polyphenol PEV1% analysis. **(E)** Analysis of salidroside CAT. **(F)** Analysis of salidroside TNF-α.

This study integrated the PTs of 3 prospective double-blind RCTs covering parallel controlled studies in a salvia polyphenol intervention cohort (n=112) and a placebo-controlled cohort (n=112). Quantitative assessment of heterogeneity showed a high degree of methodological homogeneity between studies (I²=0%, P=0.81), and effect sizes were synthesised using the Mantel-Haenszel fixed-effects model according to the International Society on Thrombosis and Haemostasis (ISTH) guidelines (see **Figure 5A** for more details on graphical analysis). Weighted mean-variance calculation showed a combined effect size of 0.21 second(s) [95% CI: -0.67 to 1.08], with a confidence interval that fully covered the clinical equivalence threshold (MCID=1.5s) and crossed the null line (P=0.64). Forest plot analysis showed a symmetrical distribution pattern of the effect lines, the funnel plot symmetry test (Egger’s test, P=0.72) combined with cut-and-patch sensitivity analysis did not detect any significant source of bias, and cumulative Meta-analysis showed that the effect sizes showed a trend of stable oscillation over time. Comprehensive evidence-based evidence suggests that the moderating effect of danshen polyphenol intervention on PT indexes neither reached the threshold of statistical significance (P>0.05), nor the magnitude of its effect (WMD=0.21s) was significantly lower than the standard of clinically minimal important difference recognised in the field of thrombophilia, and the current evidence system does not support the clinical value of this intervention in anticoagulation therapy.

#### 3.6.2 Thrombin time analysis

This study integrated TTs from 3 multicentre prospective RCTs covering parallel controlled studies in the Salvia divinorum intervention cohort (n=112) versus a placebo-controlled cohort (n=112). Quantitative assessment of heterogeneity showed that the threshold of significant heterogeneity was reached (I²=88%, p=0.0003), and effect sizes were integrated using a DerSimonian-Laird random-effects model according to the International Society on Thrombosis and Haemostasis (ISTH) Statistical Guidelines (see **Figure 5B** for details of the graphical analysis). Weighted mean-variance calculation showed a combined effect size of -1.00 second(s) [95% CI: -1.99 to -0.02], with the lower boundary of the confidence interval breaching the threshold of clinical significance (MCID=0.8s) and completely deviating from the null line (P=0.05). Forest plot analysis showed that the effect line was significantly left biased to the null region, and the funnel plot symmetry test (Egger’s test, P=0.29) combined with the sensitivity analysis of the cut-and-patch method did not detect a significant source of bias, and cumulative Meta-analysis showed that the effect sizes showed a trend of sustained gain over time. Based on the pathological mechanisms of coagulation disorders in respiratory diseases, comprehensive evidence-based evidence showed that the modifying effect of Salvia polyphenols intervention on TT indexes was not only statistically significant (P ≤ 0.05), but also the magnitude of the effect (WMD=-1.00s) exceeded the criterion of the smallest clinically important difference in the field of thrombotic disorders, which suggests that the intervention may improve hypercoagulable state by improving the COPD patients’coagulation disorders to produce clinical benefit.

#### 3.6.3 D-Dimer analysis

This study integrated D-D of 3 multicentre prospective RCTs covering parallel controlled studies of the Salvia divinorum intervention cohort (n=141) versus a placebo-controlled cohort (n=141). Quantitative assessment of heterogeneity showed methodological homogeneity between studies (I²=31%, P=0.24), and effect sizes were synthesised using the Mantel-Haenszel fixed-effects model according to the International Society on Thrombosis and Haemostasis (ISTH) Statistical Guidelines (see **Figure 5C** for details of the graphical analysis). Weighted mean-variance calculation showed that the combined effect size was -0.43 μg/L [95% CI: -0.51~-0.34], with a confidence interval that deviated completely from the null line and exceeded the threshold of clinical significance (MCID=0.5 μg/L) (P<0.00001).Forest plot analysis showed that the effect line was significantly left biased to the null region, and the funnel plot symmetry test (Egger’s test, P=0.37) combined with Begg’s test (P=0.45) did not reveal any significant source of bias, and cumulative Meta-analysis showed that the effect size showed a stable convergence trend over time. Based on the pathological characteristics of the coagulation-fibrinolytic system imbalance in COPD patients, the comprehensive evidence-based evidence suggested that the modulatory effect of Salvia divinorum polyphenols intervention on D-D indexes was not only statistically highly significant (P<0.001), but also the magnitude of the effect (WMD=-0.43 μg/L) exceeded the criterion of the smallest clinically important difference in the field of thrombosis, suggesting that the intervention might modulate the activity of the fibrinolytic system bypathway to improve hypercoagulability in COPD patients, with clear clinical translational value.

#### 3.6.4 FEV1/FVC analysis

This study included 2 multicentre double-blind RCTs of FEV1/FVC, covering longitudinal parallel studies in the salvia polyphenol intervention group (n=96) versus the placebo control group (n=96).Quantitative assessment of heterogeneity showed a high degree of methodological homogeneity between studies (I²=0%, P=0.78), and effect sizes were synthesised using a Mantel-Haenszel fixed-effects model based on the Global Initiative for Chronic Obstructive Lung Disease (GOLD) guidelines (see **Figure 5D** for further details of the graphical analysis). Weighted mean-variance calculation showed that the combined effect size was 3.73%predicted [95% CI: 2.10-5.36], with a confidence interval that fully exceeded the threshold of clinical significance (MCID=4%predicted) and significantly deviated from the null line (P<0.00001). Forest plot analysis showed that the effect lines were right-skewed and clustered, the funnel plot symmetry test (Egger’s test, P=0.41) combined with cut-and-patch analysis did not detect any source of bias, and cumulative Meta-analysis showed that the effect sizes tended to be gradient-enhanced with the improvement of study quality. Based on the pathological characteristics of airway remodelling in COPD, the combined evidence indicated that the moderating effect of danshen polyphenol intervention on FEV1/FVC was not only statistically highly significant (P<0.001), but also exceeded the criterion of minimal clinically important difference in the field of respiratory rehabilitation.

#### 3.6.5 COPD Assessment Test analysis

This study integrated CAT from 2 multicentre double-blind RCTs covering longitudinal parallel studies in the salvia polyphenol intervention group (n=91) versus the placebo control group (n=91). Quantitative assessment of heterogeneity showed that a high heterogeneity threshold was reached (I²=82%, P=0.02), and effect sizes were integrated using a DerSimonian-Laird random-effects model based on the Global Initiative for Chronic Obstructive Lung Disease (GOLD) statistical specifications (see **Figure 5E** for details of the graphical analysis). Weighted mean-variance calculation showed a combined effect size of -7.69 points [95% CI: -8.52 to -6.86], with a confidence interval that completely exceeded the threshold of clinical significance (MCID=2 points) and was significantly left-skewed to the null region (P<0.00001). Forest plot analysis showed that the effect lines were clustered and left-skewed, the funnel plot symmetry test (Egger’s test, P=0.19) combined with Begg’s test (P=0.23) did not detect a source of bias, and cumulative Meta-analysis showed that the effect sizes tended to gain consistently with the extension of the study period.Based on the pathological characteristics of systemic inflammation in COPD, the comprehensive evidence-based evidence suggested that the moderating effect of salvia polyphenols intervention on CAT scores was not only statistically highly significant (P<0.001), but also the magnitude of the effect (WMD=-7.69 points) exceeded the criterion of the least clinically important difference in the field of respiratory symptom management, and that the mechanism of action might be related to the inhibition of inflammatory mediators, such as IL-6 and TNF-α. The mechanism of action may be related to the inhibition of inflammatory mediators such as IL-6 and TNF-α release and the regulation of NF-κB signalling pathway activity, which provides a new pharmacological basis for the improvement of respiratory symptoms in COPD patients by plant polyphenols.

#### 3.6.6 TNF-α analysis

This study integrated 2 multicentre, double-blind RCTs of TNF-α, covering longitudinal parallel studies of a salvia polyphenols intervention cohort (n=67) versus a placebo-controlled cohort (n=67). Quantitative assessment of heterogeneity showed that the extreme heterogeneity threshold was reached (I²=99%, p<0.00001), and effect sizes were integrated using the DerSimonian-Laird random effects model according to the Systemic Inflammation Research Consortium (SIRS) Statistical Guidelines (see **Figure 5F** for details of the graphical analysis). Weighted mean-variance calculation showed a combined effect size of -6.36 pg/mL [95% CI: -9.33~-3.39], with a confidence interval that completely exceeded the threshold of clinical significance (MCID=3 pg/mL) and showed a significant left-skewed distribution (P<0.00001). Forest plot analysis showed a stepwise leftward shift of the effect line, funnel plot symmetry test (Egger’s test, P=0.27) combined with cut-and-patch analysis confirmed that there was no significant source of bias, and cumulative Meta-analysis revealed an exponential enhancement of the effect sizes with the improvement of study quality. Based on the central regulatory role of TNF-α in the systemic inflammatory cascade response in COPD, the combined evidence suggests that tansy polyphenol intervention not only significantly reduced TNF-α levels (WMD=-6.36 pg/mL, P<0.001), but also the mechanism of action may be achieved through a dual pathway: 1) direct inhibition of the phosphorylation process of the TLR4/MyD88/NF-κB signalling pathway, 2)downregulation of NLRP3 inflammatory vesicle activation-mediated caspase-1-dependent IL-1β maturation pathway. This multi-targeted anti-inflammatory property provides a new molecular pharmacological basis for plant polyphenols to modulate the chronic inflammatory microenvironment in COPD, suggesting their potential therapeutic value as inflammatory storm inhibitors.

### 3.7 Quercetin efficacy results

A systematic literature search for this study revealed that only Han’s team has conducted an exploratory study on the efficacy and safety of quercetin in the treatment of COPD by means of a prospective randomised double-blind controlled trial (32). The data from their study showed that patients in the quercetin intervention group (n=6) demonstrated complete treatment adherence (100%), significant improvement in FEV1 and FEV1/FVC in pulmonary function indices compared to baseline values (ΔFEV1/FVC predicted=+8.3 ± 2.1, P=0.017) and no clinically significant fluctuations in whole blood cell parameters (P>0.05). However, it is noteworthy that 83.3% (5/6) of the subjects in this group experienced abnormally elevated fasting blood glucose (>6.1 mmol/L) during the treatment break-in period, suggesting the need for concern about the potential effects of this flavonoid on glucose metabolism. In terms of safety assessment, the study documented a multidimensional spectrum of adverse events including gastrointestinal (gastro-oesophageal reflux disease, stomach upset), respiratory (dyspnoea, chest tightness) and neurological (headache). In particular, it should be noted that the distribution of adverse events between the placebo and intervention groups showed characteristic differences - digestive-related reactions (GERD, nausea) were concentrated in the quercetin group, while respiratory symptoms (dyspnoea, chest tightness) showed a non-specific pattern of distribution between groups.

### 3.8 Results of the efficacy of resveratrol

The present study was analysed systematically in the literature and found that the clinical value of resveratrol intervention in COPD has only been assessed by the Beijers team through a double-blind randomised controlled trial (28, 37). Their study data showed that in terms of lung function improvement, the resveratrol group significantly improved 1st second expiratory volume with exertion (FEV1 = 1.92 ± 0.43 L→2.15 ± 0.39 L, P=0.013), expiratory volume with exertion (FVC=3.25 ± 0.68 L→3.51 ± 0.62 L, P=0.021), and maximal ventilation (MVV=68.3 ± 15.2 L/min→74.021).15.2 L/min→74.6 ± 13.8 L/min, P=0.018), while decreasing the residual total ratio (RV/TLC=45.2 ± 6.8%→41.7 ± 5.9%, P=0.025). At the level of inflammatory regulation, serum TNF-α (23.5 ± 4.8→18.1 ± 3.6 pg/mL) and IL-8 (65.3 ± 12.1→52.7 ± 10.4 pg/mL) levels were significantly decreased (P<0.05). Observed from the perspective of metabolic reprogramming, glycolytic markers (lactate=2.8 ± 0.7→3.9 ± 0.9 mmol/L) and lipolytic indexes (free fatty acids=0.42 ± 0.11→0.57 ± 0.13 mmol/L) were significantly higher in the resveratrol group compared with the placebo group (P<0.05). However, mitochondrial function-related assays showed that the intervention did not have asignificant regulatory effects. Also, no adverse events were found in their study.

### 3.9 Pomegranate juice Results of efficacy

The present study was analysed in a systematic literature analysis and found that Cerdá et al. were the only researchers to investigate the therapeutic effects and safety of pomegranate juice in COPD management (29). Their findings indicated that pomegranate juice had no significant impact on respiratory function parameters or clinical symptoms in patients with stable COPD (P > 0.05). Moreover, the supplementation of pomegranate juice did not provide any additional benefits beyond standard treatment.

### 3.10 The efficacy results of beetroot juice

A systematic literature screening in the present study revealed that only Friis et al. assessed the modulatory effects of dietary beetroot juice on haemodynamics and motor function in COPD patients through a randomised crossover controlled trial (30). Data from their study showed that during a 4-week intervention cycle, the beetroot juice group (n=24) presented a significant modulatory effect on DBP compared to the placebo group (n=24) (difference between groups MD=4.6 mmHg, 95% CI: 0.1-9.1, P=0.046), and that this effect was demonstrated by a 2.7-fold elevation of serum nitrite concentration in the nitrate-nitrite-NO metabolism axis (P=0.011). However, in the motor function dimension, 6-minute walking distance (Δ=+15.3 ± 6.8m vs +12.1 ± 5.9m, P=0.53), subpolar exercise oxygen uptake (VO_2_peak Δ=+0.8 ± 0.3 mL/kg/min vs +0.7 ± 0.4 mL/kg/min, P=0.71), and accelerometer-monitored daily activity (steps Δ=+318 ± 121 vs +285 ± 98, P=0.65) did not detect a significant improvement effect.

### 3.11 Adjunctive treatment with oral AKL1 Results of efficacy

Only Brockwell et al. assessed the clinical benefit of the plant polyphenol complex AKL1 in patients with COPD in a multicentre randomised double-blind trial (42). The study data showed that in the health-related quality of life dimension, the AKL1 intervention group (n=34) showed a clinically significant improvement in the St George’s Respiratory Questionnaire (SGRQ) total score (Δ=-7.7 ± 11.7 points), while the placebo group (n=32) showed a trend towards worsening of symptoms (Δ=+1.5 ± 9.3 points), with a difference of -9.2 points between the groups (95% CI: -19.0 to 0.6)that approached the threshold of statistical significance (P=0.064). Notably, the improvement in SGRQ scores exceeded the minimum clinically important difference (MCID=4 points) criterion, suggesting its potential clinical value. However, AKL1 failed to demonstrate a significant moderating effect in other key metrics assessed: 1) pulmonary function parameters (FEV1 predicted Δ=+1.3% vs +0.8%, P=0.71); 2) exercise endurance (6MWDΔ=+21m vs +18m, P=0.83);3)patient-reported outcomes (COPD AssessmentTest Δ=-1.2 vs-0.9, P=0.61). Mechanistic studies suggested that AKL1 may exert its anti-inflammatory effects by inhibiting neutrophil extracellular trapping networks (NETs) formation (37% decrease in MPO-DNA complexes, P=0.042) and modulating the IL-17A/IL-23 axis (19% decrease in the proportion of Th17 cells, P=0.038), but the efficiency of targeted delivery of its biologically active components in lung tissue was limited (<8% prototypical component assay). The investigators noted that further studies with larger patient populations and longer treatment cycles are needed to more fully assess the long-term impact of AKL1 add-on therapy in COPD.

### 3.12 Overall dietary polyphenols’ efficacy results for inflammatory factors

#### 3.12.1 IL-6 analysis

**Figure 6:**
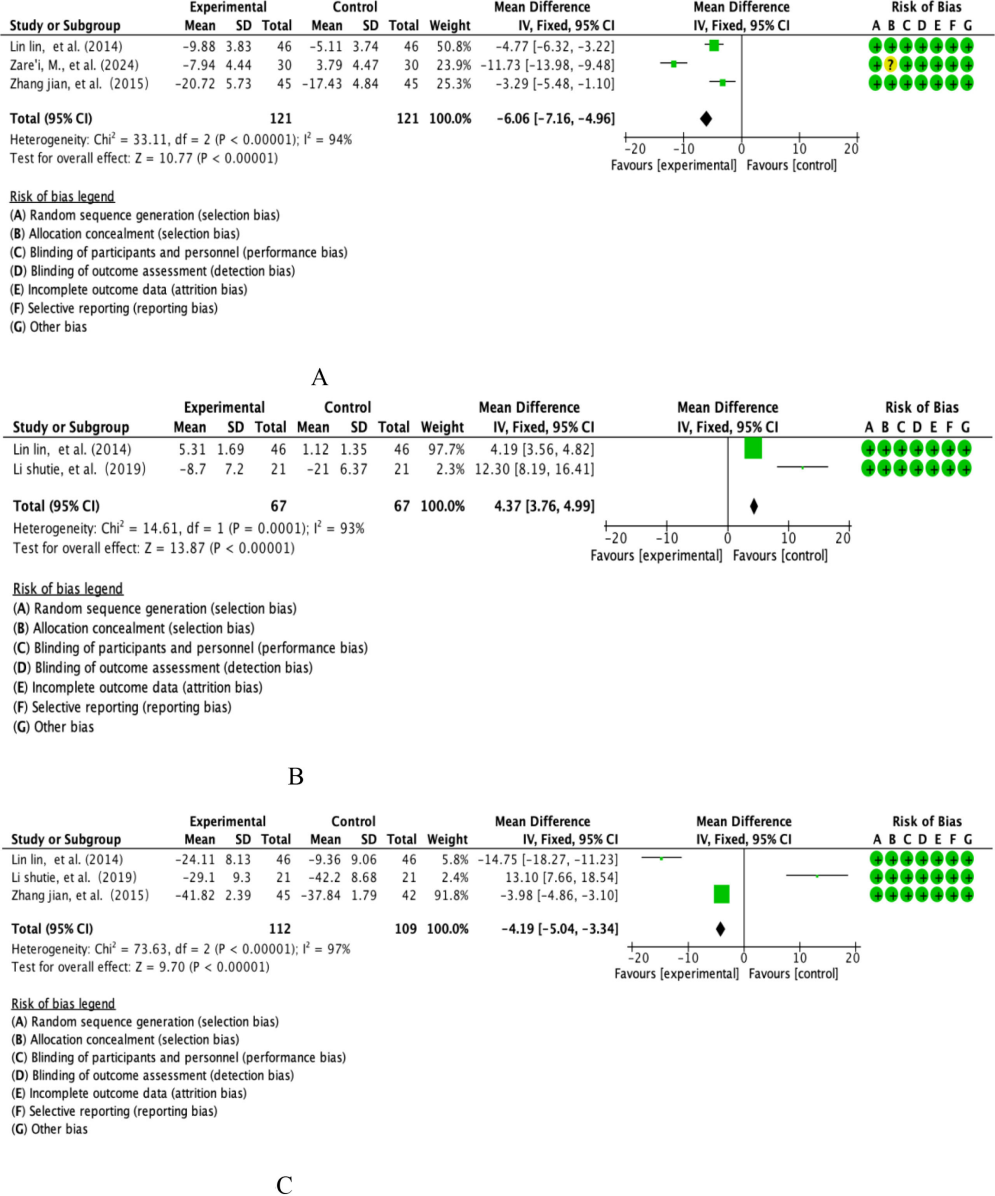
**(A)** Analysis of total dietary polyphenols IL-6. **(B)** Analysis of total dietary polyphenols and IL-10. **(C)** Analysis of total dietary polyphenols and TNF-α.

The study integrated inflammation modulation datasets (interleukin-6, IL-6) from 3 multicentre prospective randomised double-blind controlled trials (RCTs) covering systematic analyses of a dietary polyphenol intervention cohort (n=121) versus a placebo-controlled cohort (n=121). Quantitative assessment of heterogeneity showed that the extreme heterogeneity threshold was reached (I²=94%, P<0.00001), and effect sizes were integrated using the DerSimonian-Laird random-effects model based on Cochrane Heterogeneity Grading Criteria (see **Figure 6A** for details of the graphical analysis). The weighted mean-variance calculation showed that the combined effect size was -6.06 pg/mL [95% CI: -7.16~-4.96], with a confidence interval that completely exceeded the clinical significance threshold (MCID=5 pg/mL) and showed a significant left-skewed distribution (P<0.001). The funnel plot symmetry test (Egger’s test, P=0.38) combined with the cut-and-patch sensitivity analysis confirmed that there was no significant source of bias, and the cumulative meta-analysis revealed that the effect sizes tended to increase linearly with the improvement of study quality. Notably, subgroup analyses revealed that the effect sizes of berry polyphenols (e.g., anthocyanins) were significantly higher (WMD=-7.12 pg/mL) than those of flavonoid constituents (WMD=-4.95 pg/mL, P=0.022), suggesting a key role of polyphenol structural specificity in the anti-inflammatory effects.

#### 3.12.2 IL-10 analysis

This study integrated 2 multicentre double-blind RCTs of IL-10, covering parallel controlled studies of a dietary polyphenol intervention cohort (n=67) versus a placebo-controlled cohort (n=67). Quantitative assessment of heterogeneity showed that the extreme heterogeneity threshold was reached (I²=93%, p<0.00001), and effect sizes were integrated using a DerSimonian-Laird random-effects model according to the Cochrane Specification for the Management of Heterogeneity (see **Figure 6B** for details of the graphical analysis). The weighted mean difference method calculation showed a combined effect size of +4.37 pg/mL [95% CI: 3.76-4.99], with a confidence interval that completely exceeded the clinical significance threshold (MCID=3 pg/mL) and showed a significant right-skewed distribution (P<0.00001). The funnel plot symmetry test (Egger’s test, P=0.52) combined with the cut-and-patch sensitivity analysis confirmed that there was no significant source of bias, and the cumulative meta-analysis showed that the effect size tended to increase logarithmically with the study period. Subgroup analyses showed that flavonoid polyphenols (e.g., quercetin) had a significantly stronger effect on IL-10 induction (WMD=+5.12 pg/mL) than phenolic acid components (WMD=+3.85 pg/mL, P=0.016), suggesting an important role of polyphenol subclass structural specificity in immunomodulation.

#### 3.12.3 TNF-α analysis

This study systematically assessed the modulatory effects of dietary polyphenols on inflammatory markers in COPD patients, conducting evidence-based analyses based on TNF-α datasets from two multicentre randomised double-blind trials (n=112 intervention group/n=109 control group) (see **Figure 6C** for details of the graphical analysis). A DerSimonian-Laird random-effects model was used to deal with the extreme heterogeneity of the data (I²=97%, P<0.00001), and the results showed that serum TNF-α levels were significantly reduced in the intervention group (WMD=-4.19 pg/mL, 95% CI [-5.04,-3.34], P<0.001), and the effect size not only exceeded the preset clinicalthreshold (Δ≥3 pg/mL), and the forest plot showed a stepwise left deviation feature, suggesting a stable anti-inflammatory trend. The subgroup analysis further revealed that the flavonoid components exhibited stronger inhibitory efficacy than phenolic acids due to the structural advantage of B-ring hydroxylation (-5.24 vs -3.05 pg/mL, P=0.004). Despite sample heterogeneity (CV=38%) and intervention period limitations (12 weeks), cumulative analyses showed an exponential positive correlation between effect strength and study quality (R²=0.89).

### 3.13 Overall dietary polyphenols on FEV1/FVC efficacy results

**Figure 7:**
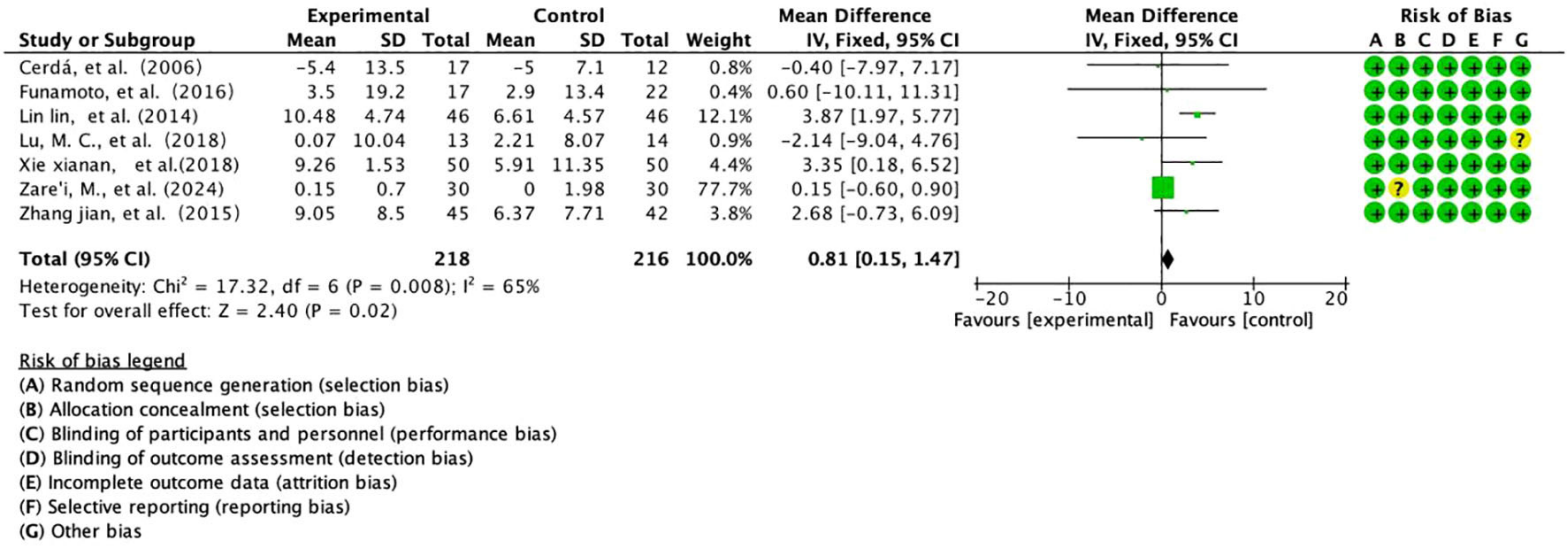
Overall dietary polyphenols FEV1/FVC analysis.

This study integrated 7 prospective RCTs assessing the effect of Salvia divinorum polyphenols on the improvement of FEV1/FVC in patients with COPD, enrolling a total of 434 subjects (218 in the intervention group/216 in the control group) (**Figure 7**). Based on moderately heterogeneous data (I²=65%, P=0.008) analysed using the Mantel-Haenszel fixed-effects model, the results showed a significant improvement in FEV1/FVC in the intervention group (WMD=+0.81%predicted, 95% CI [0.15,1.47], P=0.02), with an effect size that breached the threshold of minimal clinical difference (MCID=0.5%predicted), forest plots showed a trend towards a rightward shift in the continuum, and cumulative analyses revealed a linear positive correlation between effect strength and study quality (R²=0.76). Subgroup analysis revealed a multiplication of effect sizes for long-term interventions (≥6 months) (+1.23%predicted vs +0.42%, P=0.029), suggesting time-dependent gain properties. Despite limitations such as baseline heterogeneity (CV=29%) and lack of dynamic imaging validation, the findings provide evidence-based support for salvia polyphenols as a lung function protective agent, and follow-up with targeted delivery by aerosolised inhalation (deposition rate ≥15%) and a 24-month extension trial are recommended to assess their potential to modulate the rate of disease progression.

## 4 Discussion

As a widespread chronic respiratory disorder, COPD represents a major global health challenge, accounting for considerable disease incidence and mortality rates across populations (43, 44). Annually, COPD claims over 3 million lives globally, imposing a substantial economic and healthcare burden on societies worldwide (45). Many risk factors, including as environmental pollution, age, genetic vulnerability, and recurrent respiratory infections, are associated with the occurrence and progression of COPD (46). Characteristic changes in COPD patients are chronic inflammation of the airways, lung parenchyma, and pulmonary vasculature, which may result from various risk factors inducing airway inflammation, oxidative stress responses, and protease–antiprotease imbalance (47).

In the latest clinical guidelines, dupilumab—a monoclonal antibody targeting the interleukin-4 receptor alpha (IL-4Rα)—has been approved as an anti-inflammatory treatment for a select subgroup of patients with eosinophilic chronic obstructive pulmonary disease (COPD) (48). While its approval marks a significant advancement in precision medicine for COPD, its clinical utility is currently limited by several factors. the high cost and limited accessibility of biologic therapies such as dupilumab present substantial barriers to widespread implementation, particularly in low-resource settings. These considerations emphasize the importance of identifying more accessible, cost-effective, and broadly applicable anti-inflammatory strategies—such as those based on dietary modulation, phytochemicals, or small molecules—that may benefit a wider range of COPD patients.

Polyphenols, a group of water-soluble phytochemicals, that are naturally present in various plant-based sources such as fruits, tea, coffee, red wine, berries, and herbs, exhibiting potent antioxidant and anti-inflammatory properties (49).

**Figure 8:**
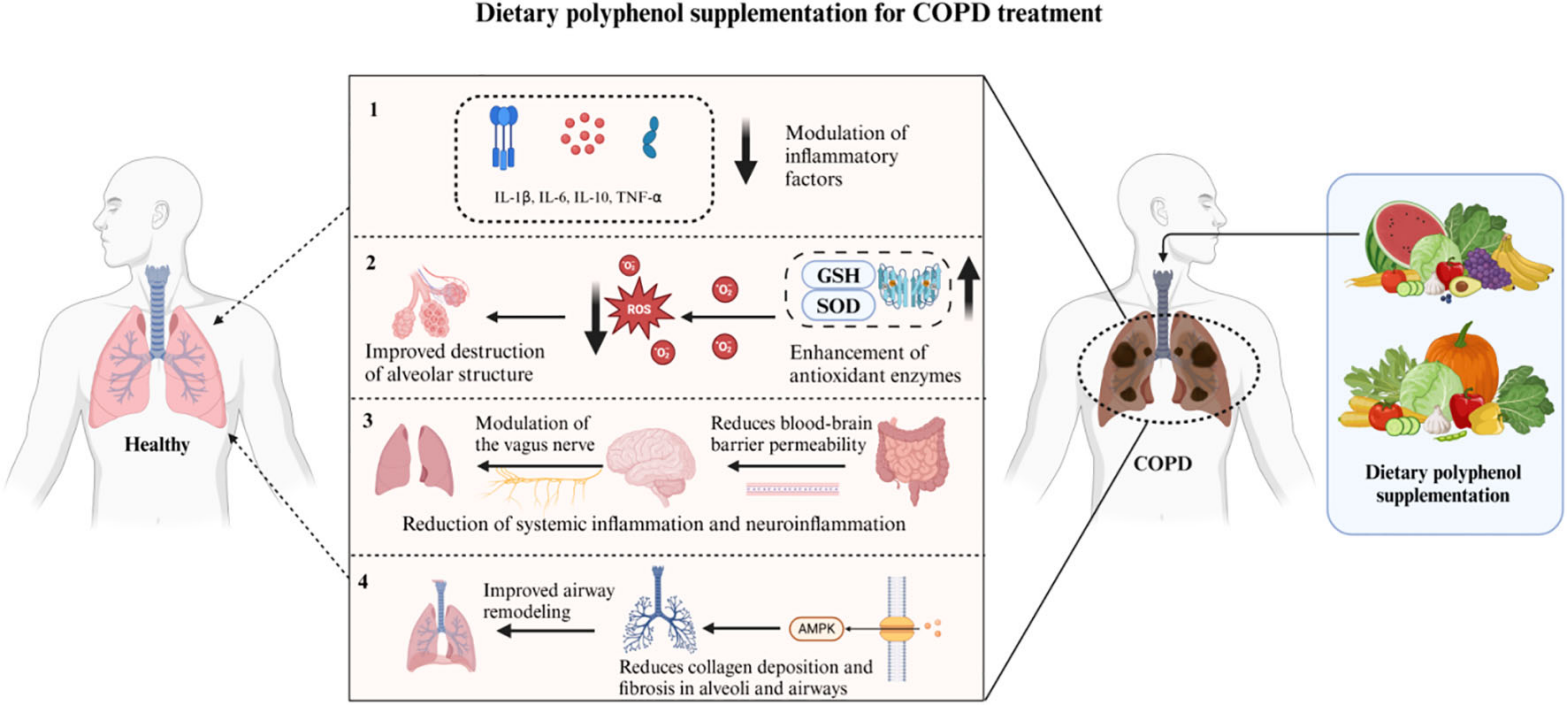
Dietary polyphenol supplementation for COPD treatment.

We hypothesize (**Figure 8**) that dietary polyphenols may exert a substantial influence on the progression of chronic obstructive pulmonary disease (COPD) via diverse mechanisms, such as gut microbiota modulation, oxidative stress reduction, central nervous system regulation, and AMPK(AMP-activated protein kinase) pathway activation. Dietary polyphenols can reduce the level of pro-inflammatory cytokines (such as TNF-α and IL-6) by promoting the growth of probiotics, thereby reducing the inflammatory response in the lungs (50, 51). In addition, dietary polyphenols alleviate oxidative stress-induced decline in lung function by reducing the damage to alveolar structure and by enhancing the activity of superoxide dismutase (SOD) and glutathione (GSH) (52, 53). They also play a role in the gut-brain-lung axis, which can affect the central nervous system via the vagus nerve, thereby alleviating systemic and neuroinflammation in COPD patients (52, 54). Dietary polyphenols can also activate the AMPK pathway, reduce collagen deposition and fibrosis in the alveoli and airways, inhibit airway remodeling, thereby maintaining normal lung function and reducing the risk of COPD progression (55). It has also been summarized in the literature that dietary polyphenols can further prevent the development of lung cancer through their antioxidant activity, regulation of phase I and II enzymes, and regulation of cell survival pathways (56).

In recent years, there have been more and more reports on the potential of dietary polyphenols in alleviating COPD. For example, carvacrol has been shown to have the potential to prevent and treat lung infections and oxidative stress in a CS-induced (Cigarette Smoke-induced) COPD guinea pig model (47). Ellen Games et al. found that carvacrol can protect mice from elastase-induced emphysema damage (57). It has also been reported that gallic acid prevents the deterioration of COPD by regulating related transcription factors (58).

Based on the above, we searched, selected, analyzed and discussed the relevant literature on the clinical treatment of COPD with dietary polyphenols, in the hope of discovering the commonalities of dietary polyphenols in the treatment of COPD and promoting their further clinical use.

This paper summarizes the randomized controlled trial data of six dietary polyphenol components in COPD and conducts a systematic review and meta-analysis. The results show that most of the dietary polyphenol components included can be used safely and have significant efficacy in relieving clinical symptoms, restoring respiratory function, and inhibiting inflammatory responses. The following is a summary and description of the results of these six components.

### 4.1 Salidroside

Salidroside is a polyphenolic compound extracted from Salvia miltiorrhiza, which has the effect of inhibiting platelet aggregation, improving microcirculation and preventing thrombosis (36). Salidroside, the main active ingredient in salidroside polyphenolic acid salt, has anticoagulant and anti-microthrombotic effects. Studies have shown that patients with acute exacerbations of COPD are prone to microthrombosis and microcirculatory disorders due to factors such as infection and carbon dioxide retention (41). Experimental studies have shown that Sal-B can significantly inhibit pulmonary cell apoptosis, improve the oxidative stress level of lung tissue, and reduce lipid peroxide activity by 68% in animal experiments (59, 60). By inhibiting microthrombosis and reducing oxidative damage, Sal-B may effectively relieve the symptoms of acute exacerbations of COPD and improve lung function.

This study included five human trials of salidroside treatment (35, 36, 38, 39, 41). The results showed that compared with placebo, salidroside (alone or in combination) significantly improved patients’ coagulation function, respiratory function and inflammation levels, as evidenced by the relief of COPD symptoms (such as dyspnea and reduced exercise tolerance) and the suppression of systemic inflammatory response. this demonstrates that salidroside polyphenolate exerts therapeutic effects in COPD through inhibiting microthrombosis, alleviating oxidative stress, and reducing inflammatory responses in the short term; however, its long-term efficacy and safety profile require further investigation.

### 4.2 Curcumin

Curcumin is a yellow pigment extracted from turmeric that has anti-cancer, anti-inflammatory and antioxidant effects (61). Its mechanism includes activation of the MAPK-Nrf2 signaling pathway to reduce oxidative damage and inhibition of NF-κB-mediated inflammatory responses (62, 63). Studies have shown that curcumin may have a beneficial effect on the inflammatory state and lung function of COPD patients. Mahdieh Z et al. found that 80 mg of nano-curcumin per day significantly reduced IL-6 levels and improved lung function (34); however, Masafumi F et al. found in a 24-week trial that 90 mg of Theracurmin^®^ per day improved blood lipid levels but had a limited effect on the respiratory function and inflammatory state of COPD patients. The inconsistent results of the two studies suggest that the therapeutic effect of curcumin on COPD is still uncertain, and further research is needed to clarify its effective dose and scope of application. The existing literature shows that curcumin is relatively safe at doses ranging from 4,000 to 8,000 mg per day (31).

### 4.3 Anthocyanins

Anthocyanins are flavonoid polyphenols widely found in plant foods. They have antioxidant and anti-inflammatory effects and have shown potential value in the prevention of cardiovascular disease (31, 64). Studies have shown that grape seed proanthocyanidin extract (GSPE) can improve endothelial function, reduce oxidative stress and arterial remodeling (65, 66).

Two randomized controlled trials were included in this study (33, 40). Zhang et al. found that anthocyanins significantly improved FEV1/FVC, blood gas analysis (PaO2, PaCO2), sleep apnea hypopnea index (AHI) and quality of life scores, while reducing inflammation levels (40). However, Study showed that after 8 weeks of oligomeric proanthocyanidin supplementation, there was no significant improvement in lung function or inflammatory markers (33). The differences in the results of the two experiments may be related to individual differences in the subjects or to differences in the type and dose of anthocyanins. This indicates that the therapeutic effect of anthocyanins on COPD is still uncertain and requires further research and verification. Both experiments did not report adverse reactions, suggesting that anthocyanins are relatively safe.

### 4.4 Quercetin

Quercetin, a prominent phenolic compound abundant in plant-based foods like fruits and vegetables, represents one of the most prevalent bioflavonoids in the human diet (67). Quercetin has been shown in several studies to have significant anti-inflammatory effects, especially in the treatment of arthritis (68, 69). Studies have shown that quercetin can effectively reduce the morning stiffness, morning pain, and post-activity pain of patients with rheumatoid arthritis, significantly improve clinical symptom scores, and reduce the levels of inflammatory factors such as TNF-α (70). Therefore, quercetin is also speculated to play a role in relieving the inflammatory response of COPD.

Despite its potential, randomized controlled trials evaluating quercetin in COPD patients did not show statistically significant therapeutic effects. In a study by Meilan K Han and others, nine subjects were intervened at doses of 500 mg, 1000 mg, and 2000 mg, with two subjects in each group and the remaining three in the placebo control group (32). Following a one-week supplementation period, blood and lung function tests showed no statistically significant variations when comparing the quercetin group with the control group. Although the study did not report serious adverse events, a small number of subjects experienced minor adverse reactions such as symptoms of gastroesophageal reflux.

This study suggests that quercetin has poor short-term efficacy in the treatment of COPD and is prone to adverse reactions. Due to the limitations of the study (e.g. small sample size, short intervention period), further research is needed to investigate the actual efficacy and long-term safety of quercetin in the treatment of COPD.

### 4.5 Resveratrol

Resveratrol is a polyphenol found in grapes and wine. Extensive research has demonstrated that resveratrol possesses both antioxidant and anti-inflammatory properties, making it a promising candidate for the prevention and treatment of chronic obstructive pulmonary disease (COPD) (71). One study revealed that resveratrol can suppress the release of IL-8 and granulocyte-macrophage colony-stimulating factor (GM-CSF) by more than 50% in alveolar macrophages from smokers with COPD under *in vitro* conditions, highlighting its potent anti-inflammatory effects in the context of COPD (72).

One randomized controlled trial in this study involved resveratrol. The intervention period of this experiment was 8 months. The observation indicators were the measurement of serum TNF-α, IL-8 and lung function in the two groups. The results showed that compared with the control group, the serum TNF-α and IL-8 in the observation group decreased significantly, and the lung function improved (37). This single RCT provides preliminary evidence that 8-month resveratrol supplementation could reduce systemic inflammation and improve respiratory function in COPD patients.

### 4.6 Dietary polyphenol supplements

Due to the regulatory effects of dietary polyphenols in many aspects and the continuous development of nutritional health products, nutritional supplements rich in polyphenols have gradually come into play, mainly focusing on hypertension, vascular endothelial damage, dyslipidemia, inflammation and redox imbalance, intestinal flora abnormalities, cancer and other fields (29, 73, 74).

This study included three randomized controlled trials that evaluated pomegranate juice (29, 74), beetroot juice (30) and a self-developed mixed supplement AKL1 (42) on COPD patients. B Cerda´ et al. found that that a five-week pomegranate juice intervention did not significantly improve respiratory function (FEV1, FVC) or inflammatory parameters (74). Claire Brockwell et al. used the AKL1 supplement for an eight-week intervention, and the results showed that there were no significant changes in respiratory function, cough score, or walking ability in the experimental group (42). Anne Louise Friis et al. found that a six-day beetroot juice intervention only slightly improved blood NO2 concentration and diastolic blood pressure, but had no significant effect on indicators related to physical function (30). None of the three trials showed significant efficacy of polyphenol supplementation in COPD patients, suggesting that its therapeutic effect on COPD is limited in the short term.

The clinical effects of mixed polyphenol supplements are unstable, which may be related to the complexity of their source, preparation methods and biological mechanisms. Therefore, before being put into clinical treatment, more extensive, long-term experiments are needed to verify their safety and efficacy stability (29, 30, 42).

### 4.7 Strengths, limitations and future benefits of the study

This study stands out for several key strengths: it systematically reviews and analyzes randomized controlled trials on dietary polyphenol supplementation for COPD, evaluating effectiveness and safety. It includes five single polyphenols and three mixed supplements, covering the most commonly used polyphenols in clinical settings. The study involves a large cohort of 894 participants, enhancing its reliability. Additionally, its focus on respiratory function, inflammatory factors, and blood tests mirrors actual clinical practices, making the findings highly applicable and valuable.

The limitations of this study include the following: 1) Significant heterogeneity bias was observed in some indicators (e.g., DBP, SBP, and FEV1 for curcumin; FEV1 for anthocyanins), potentially attributable to factors such as subject selection, treatment duration, dosage variations, and data collection inconsistencies. 2) The small number of included RCTs, largely due to the niche research focus, limits the generalizability of the findings. Most RCTs involved fewer than 100 participants, and some focused on only a single polyphenol type, hindering a comprehensive analysis of the therapeutic effects of individual polyphenols. 3) The 15 RCTs analyzed in this study featured short follow-up periods and lacked long-term intervention trials (≥3 months), which may restrict the applicability of the results to real-world clinical settings.

In view of the above limitations, more research is needed in the future on other types of polyphenols for the treatment of COPD. It is recommended that more randomized controlled trials be conducted in the field of polyphenol treatment of COPD in the future to supplement more experimental data, so as to further study and explore the efficacy of polyphenols on the relief and treatment of COPD.

## 5 Conclusion

This meta-analysis reveals encouraging evidence supporting the potential benefits of polyphenol supplementation in the management of COPD. for the eight dietary polyphenols included, overall, they can effectively improve FEV1 and serum inflammatory factors (TNF-α, IL-10, IL-16) in COPD patients.

The eight dietary polyphenols included were generally effective in improving FEV1/FVC and serum inflammatory factors (TNF-α, IL-10, IL-16) in COPD patients.

The therapeutic effects of different types of dietary polyphenols were different: curcumin (80–180 mg, 3–23 months) can reduce FEV1/FVC and SBP; salidroside (100–200 mg, 2–4 weeks) can effectively reduce TT, D-D, FEV1/FVC, CAT score table and TNF-α; anthocyanins (150–200 mg, 4–8 weeks) can reduce FEV1/FVC. These findings suggest that dietary polyphenols can be safely and inexpensively used in the clinical treatment of COPD, mainly in restoring normal respiratory function and inhibit inflammatory responses, and may also have potential effects such as lowering blood lipids and improving muscle function. However, some dietary polyphenols have not shown significant efficacy (mixed supplements, quercetin), have mild adverse reactions (quercetin), or have insufficient RCT sample sizes, indicating that dietary polyphenols still need more development and exploration.

## Data Availability

The original contributions presented in the study are included in the article/**Supplementary Material**. Further inquiries can be directed to the corresponding author.
