# Supplementary figures and images for: Efficacy and safety of dietary polyphenol supplements for COPD: a systematic review and meta-analysis
Source: Front Immunol. 2025 Jul 23;16:1617694. doi: 10.3389/fimmu.2025.1617694 (PMC12325041; doi:10.3389/fimmu.2025.1617694)

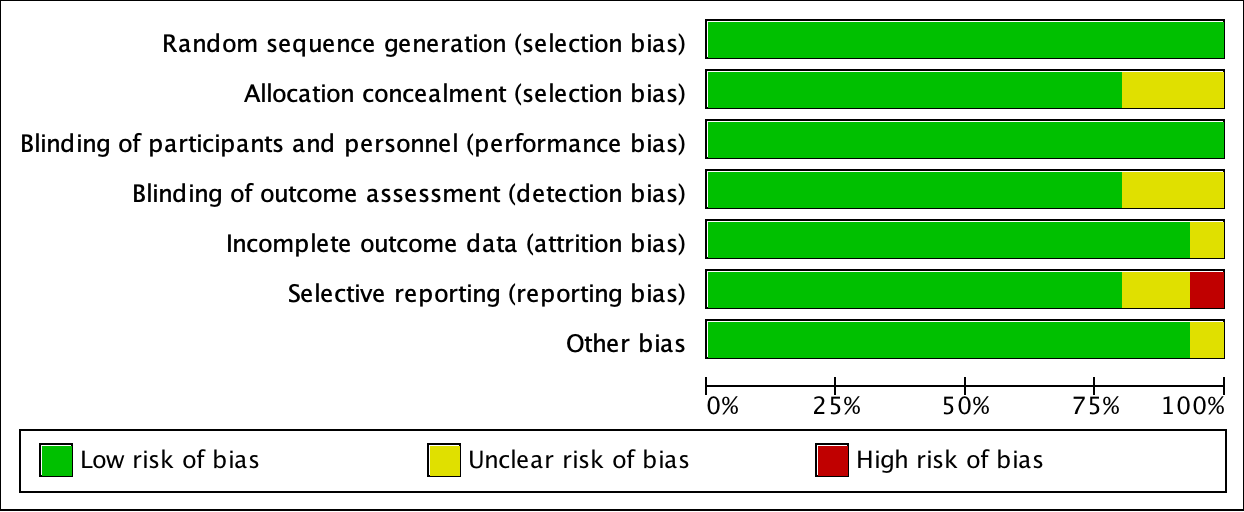

Supplement: Supplementary file 1 [file DataSheet1.zip › 3.31Efficacy and safety of dietary polyphenol supplements for COPD a systematic review and meta-analysis/(A)Risk of bias.png]

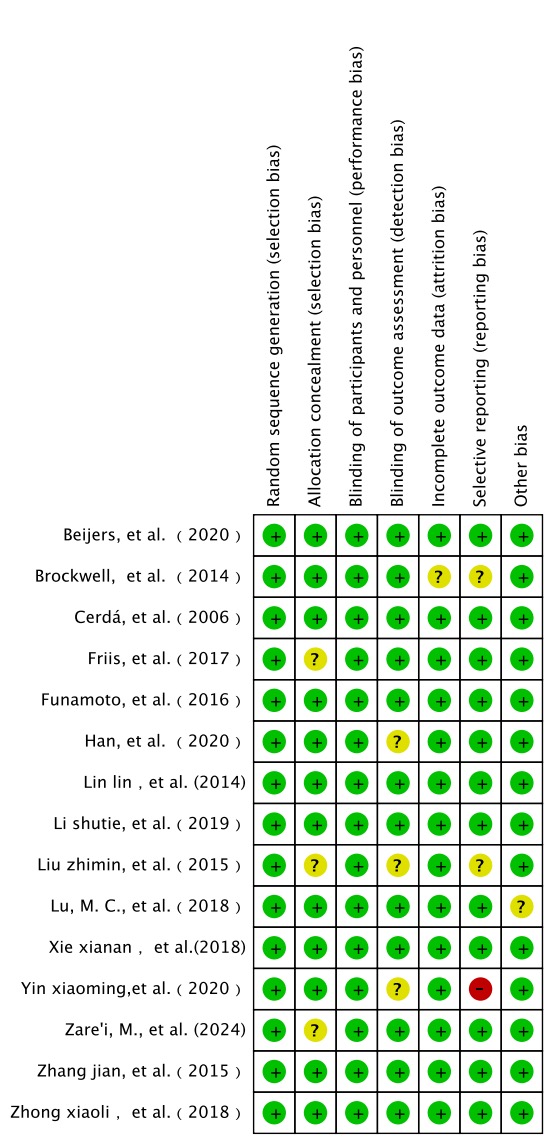

Supplement: Supplementary file 1 [file DataSheet1.zip › 3.31Efficacy and safety of dietary polyphenol supplements for COPD a systematic review and meta-analysis/(B) Risk of bias summary.jpeg]

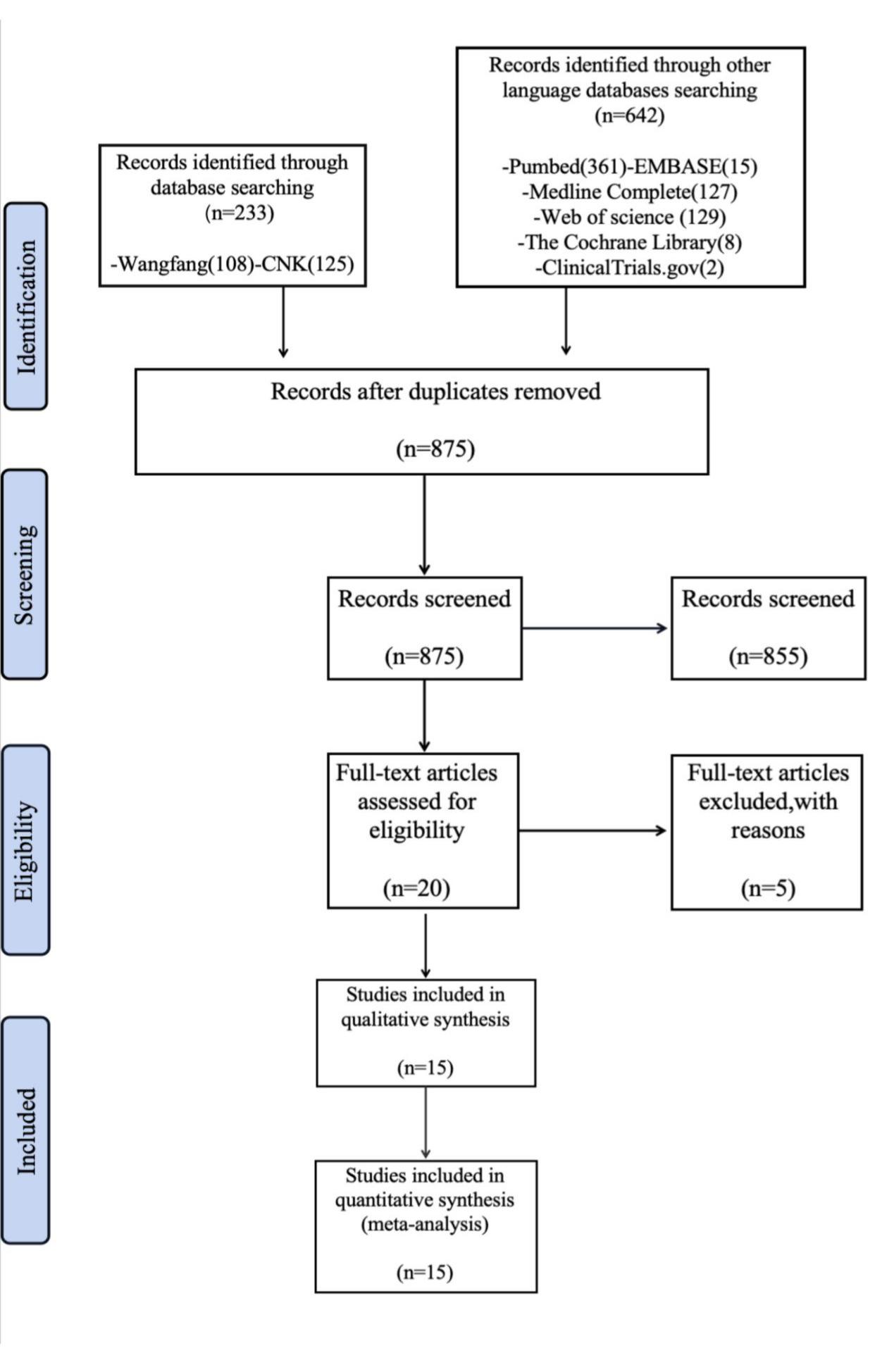

Supplement: Supplementary file 1 [file DataSheet1.zip › 3.31Efficacy and safety of dietary polyphenol supplements for COPD a systematic review and meta-analysis/Figure 1 Flow diagram.jpeg]

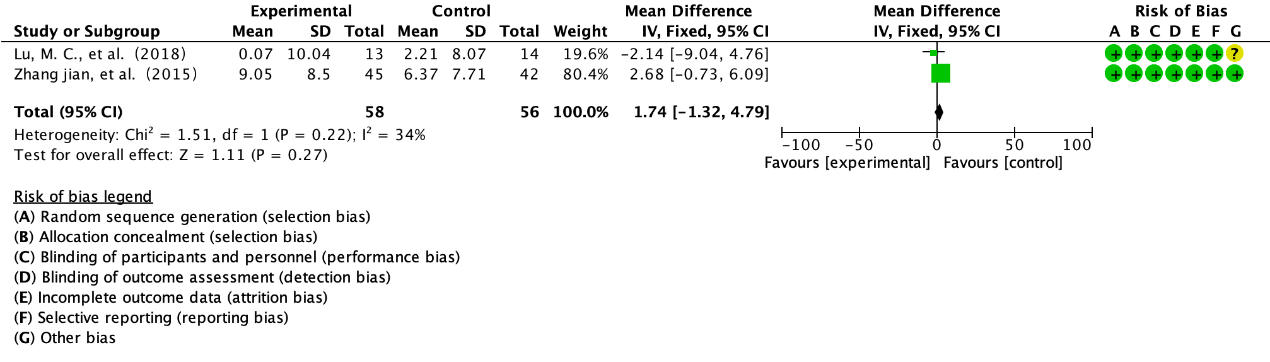

Supplement: Supplementary file 1 [file DataSheet1.zip › 3.31Efficacy and safety of dietary polyphenol supplements for COPD a systematic review and meta-analysis/Figure 4A Analysis of anthocyanin FEV.png]

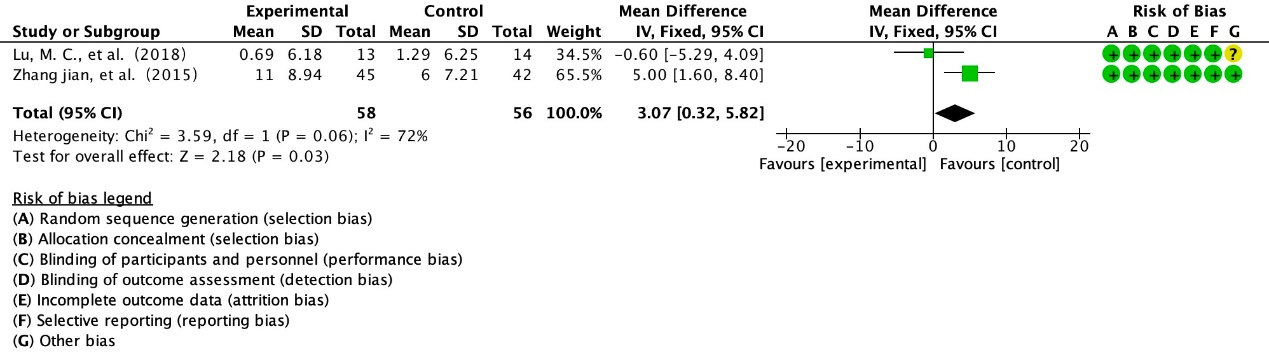

Supplement: Supplementary file 1 [file DataSheet1.zip › 3.31Efficacy and safety of dietary polyphenol supplements for COPD a systematic review and meta-analysis/Figure 4B Analysis of anthocyanin FEV1FVC.jpeg]

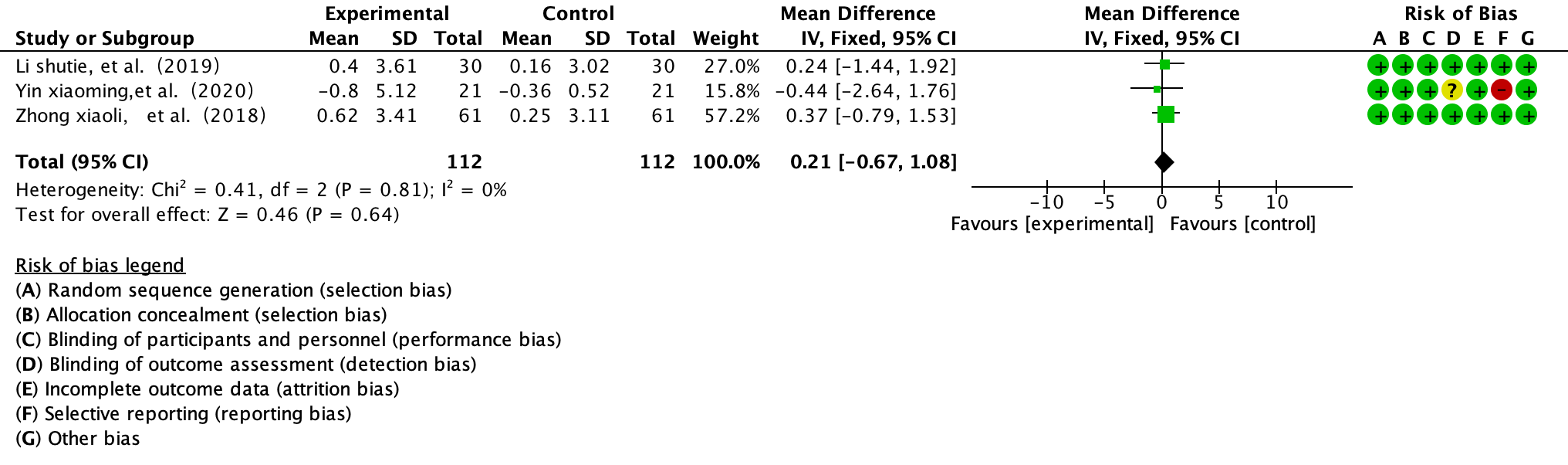

Supplement: Supplementary file 1 [file DataSheet1.zip › 3.31Efficacy and safety of dietary polyphenol supplements for COPD a systematic review and meta-analysis/Figure 5A Salvia polyphenol PT analysis.png]

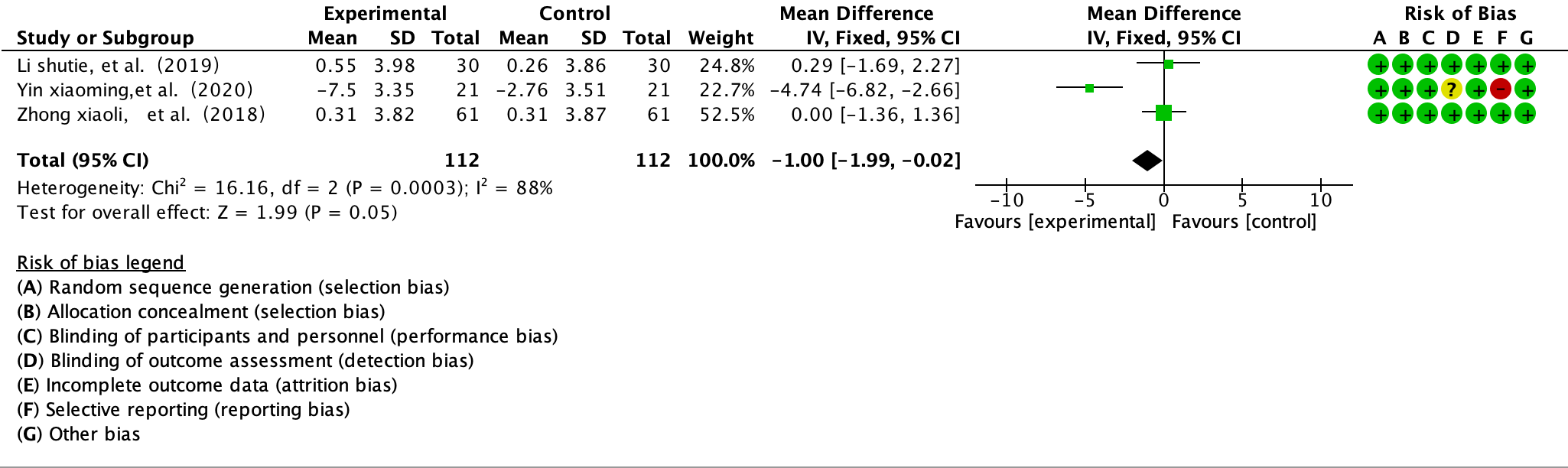

Supplement: Supplementary file 1 [file DataSheet1.zip › 3.31Efficacy and safety of dietary polyphenol supplements for COPD a systematic review and meta-analysis/Figure 5B Salvia polyphenol TT analysis.png]

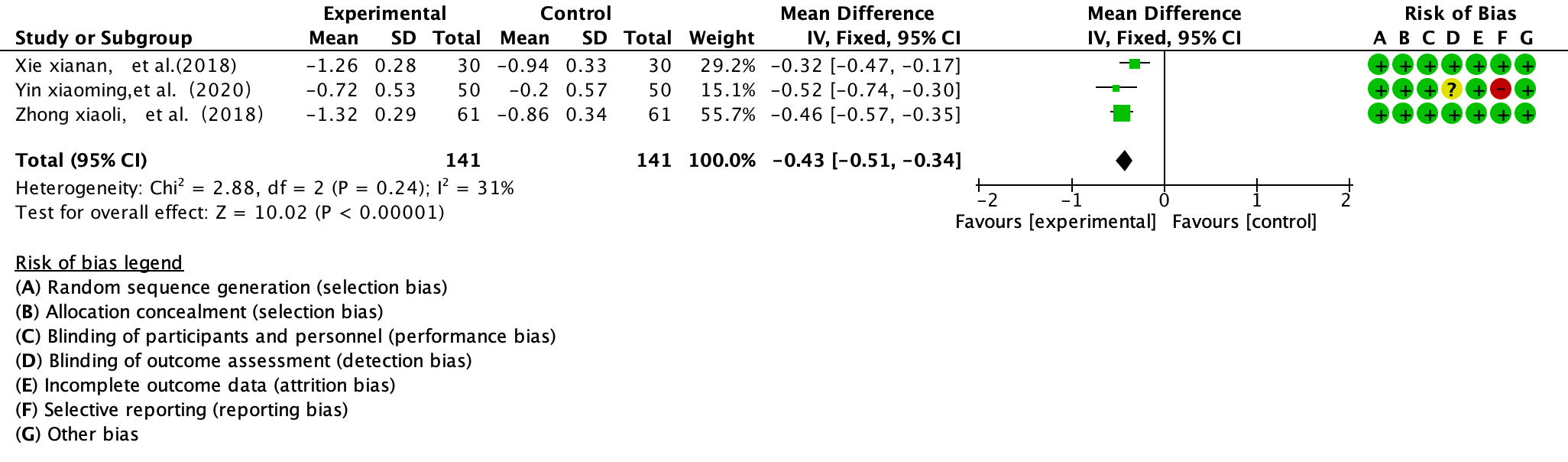

Supplement: Supplementary file 1 [file DataSheet1.zip › 3.31Efficacy and safety of dietary polyphenol supplements for COPD a systematic review and meta-analysis/Figure 5C Salvia polyphenol D-D analysis.png]

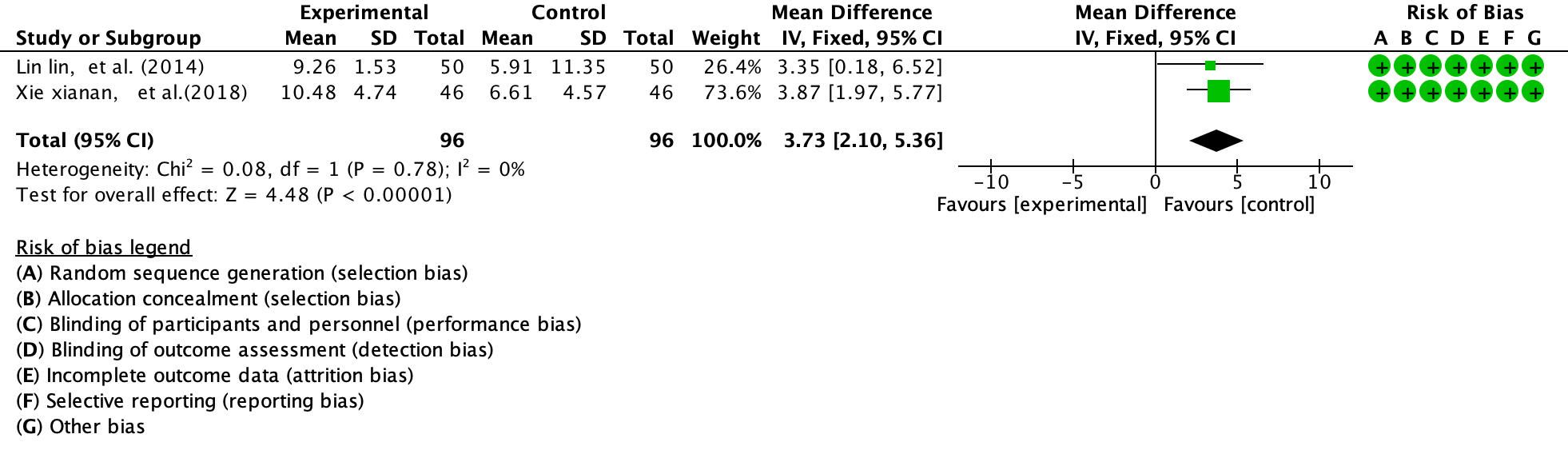

Supplement: Supplementary file 1 [file DataSheet1.zip › 3.31Efficacy and safety of dietary polyphenol supplements for COPD a systematic review and meta-analysis/Figure 5D Salvia polyphenol PEV1% analysis.png]

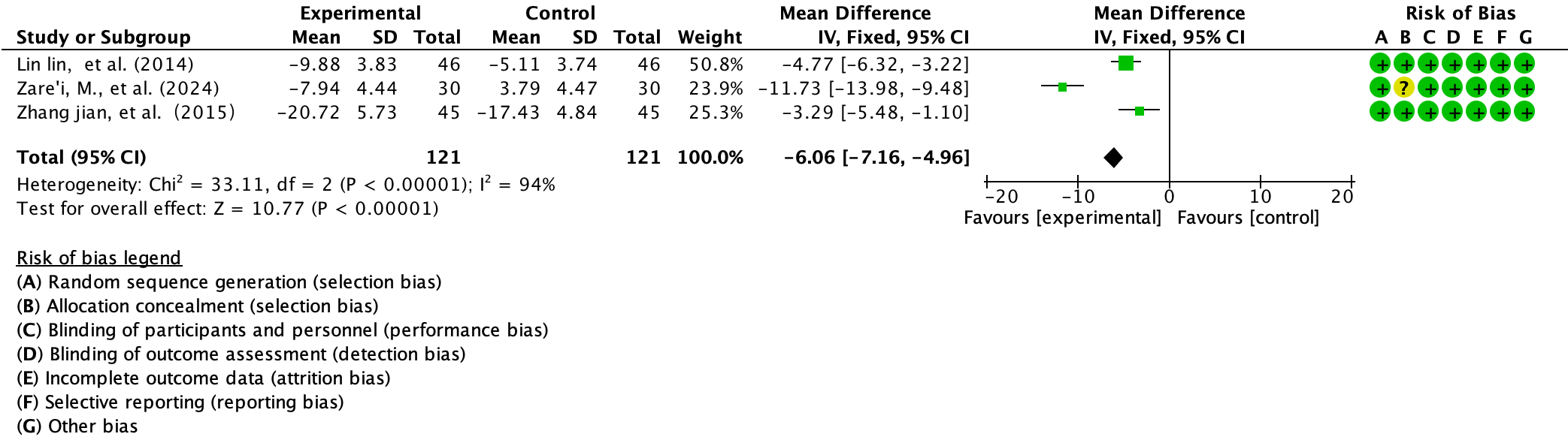

Supplement: Supplementary file 1 [file DataSheet1.zip › 3.31Efficacy and safety of dietary polyphenol supplements for COPD a systematic review and meta-analysis/Figure 6A Analysis of total dietary polyphenols IL-16.png]

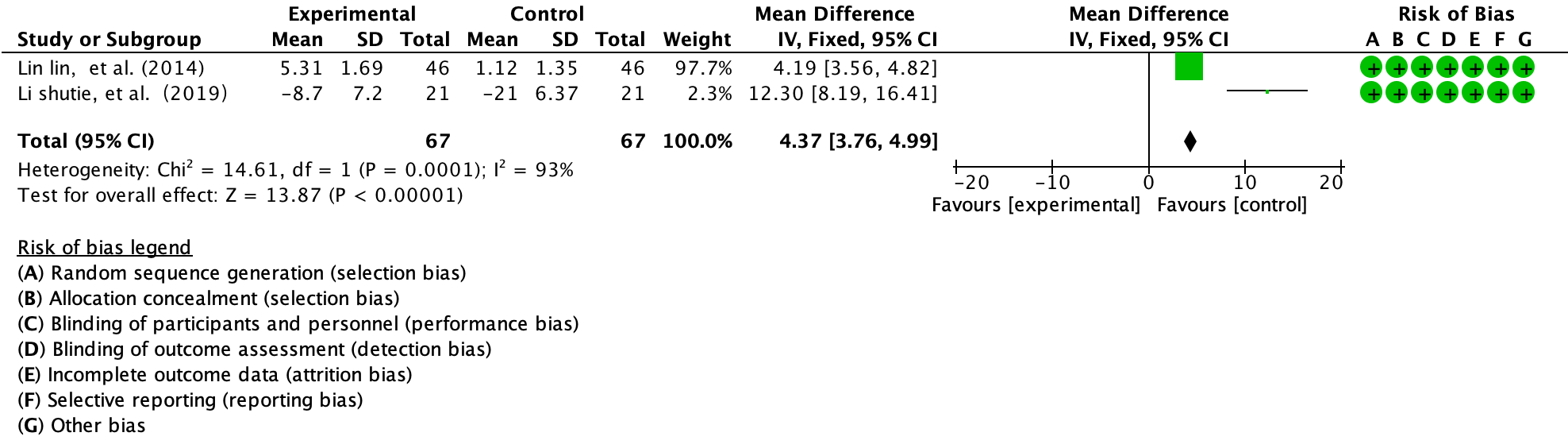

Supplement: Supplementary file 1 [file DataSheet1.zip › 3.31Efficacy and safety of dietary polyphenol supplements for COPD a systematic review and meta-analysis/Figure 6B Analysis of total dietary polyphenols and IL-10.png]

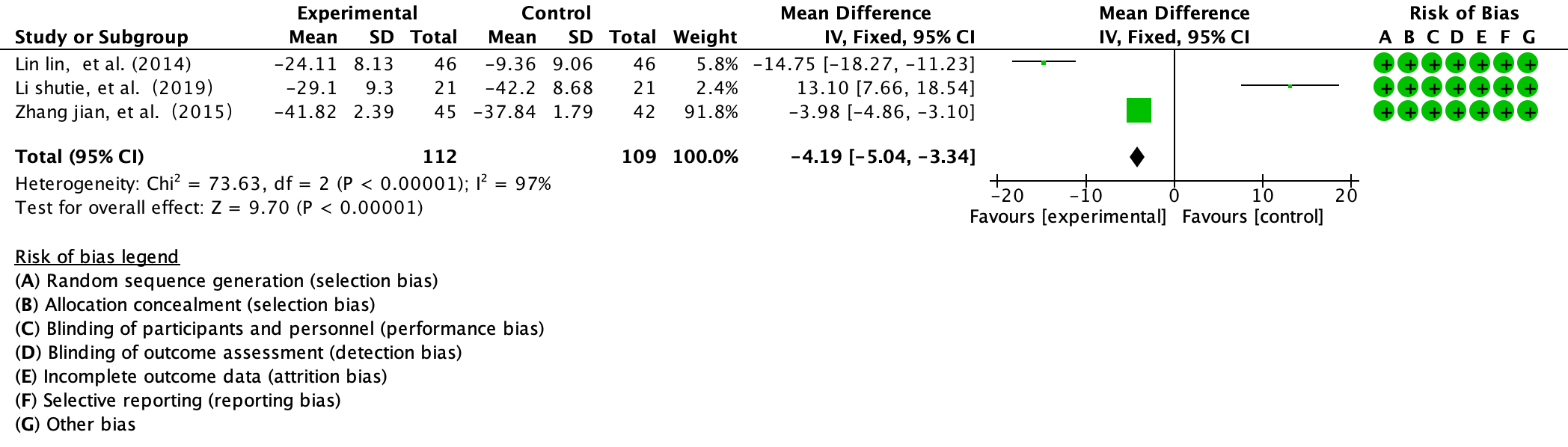

Supplement: Supplementary file 1 [file DataSheet1.zip › 3.31Efficacy and safety of dietary polyphenol supplements for COPD a systematic review and meta-analysis/Figure 6C Analysis of total dietary polyphenols and TNF-α.png]

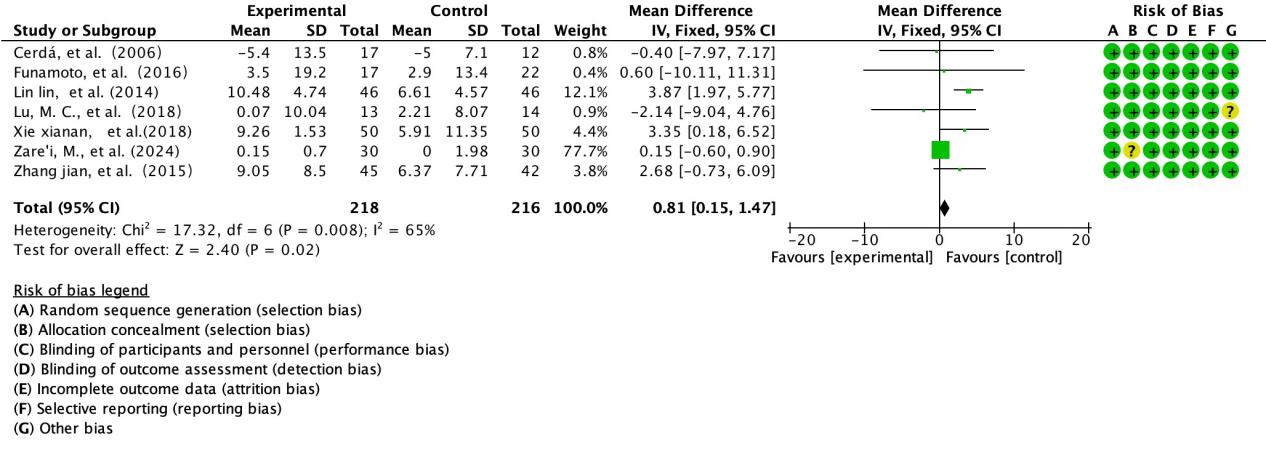

Supplement: Supplementary file 1 [file DataSheet1.zip › 3.31Efficacy and safety of dietary polyphenol supplements for COPD a systematic review and meta-analysis/Figure 7 Overall dietary polyphenols FEV1% analysis.jpeg]

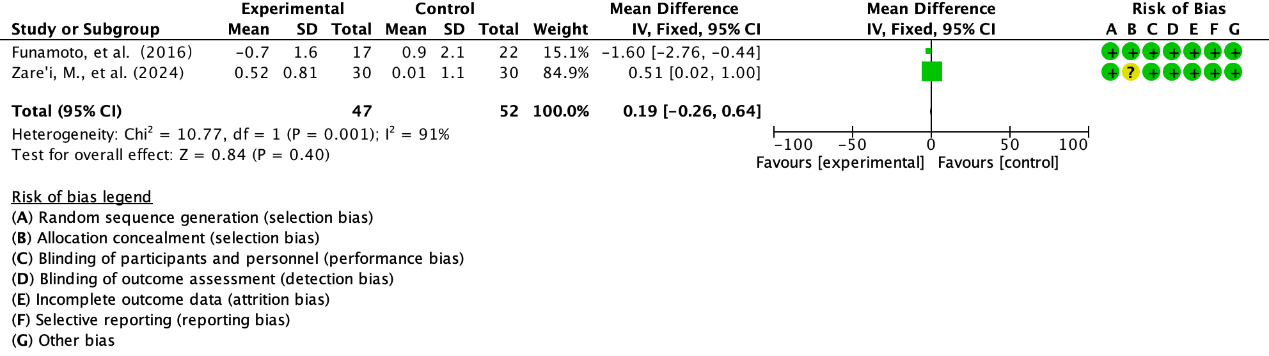

Supplement: Supplementary file 1 [file DataSheet1.zip › 3.31Efficacy and safety of dietary polyphenol supplements for COPD a systematic review and meta-analysis/Figure3A Curcumin body weight index analysis.png]

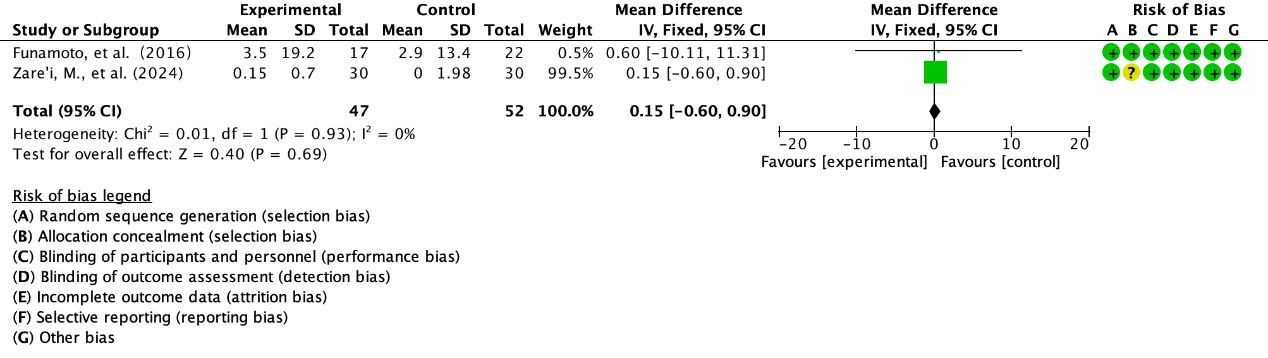

Supplement: Supplementary file 1 [file DataSheet1.zip › 3.31Efficacy and safety of dietary polyphenol supplements for COPD a systematic review and meta-analysis/Figure3B Curcumin diastolic blood pressure analysis.png]

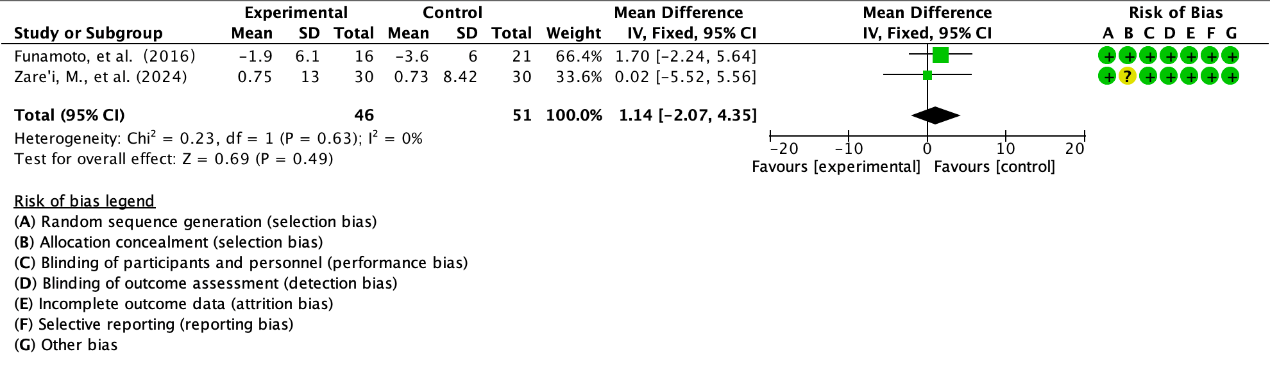

Supplement: Supplementary file 1 [file DataSheet1.zip › 3.31Efficacy and safety of dietary polyphenol supplements for COPD a systematic review and meta-analysis/Figure3C Curcumin systolic blood pressure analysis.png]

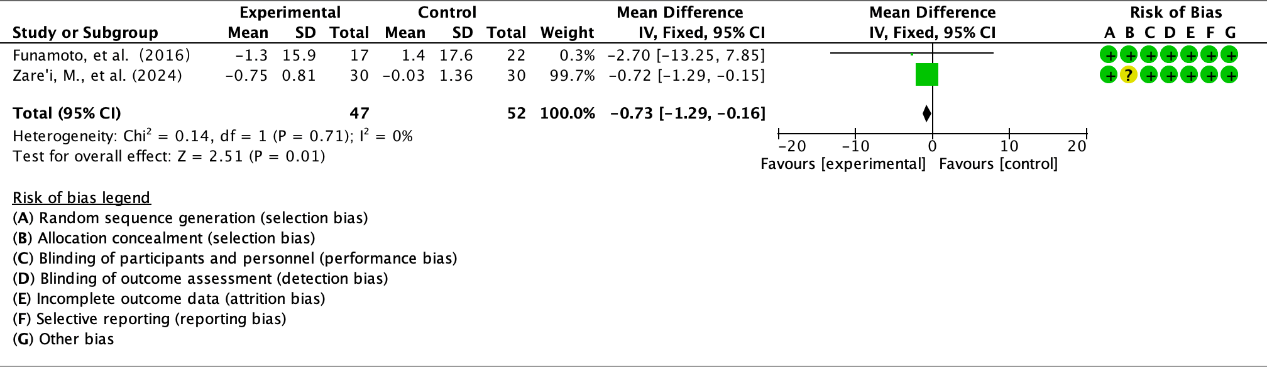

Supplement: Supplementary file 1 [file DataSheet1.zip › 3.31Efficacy and safety of dietary polyphenol supplements for COPD a systematic review and meta-analysis/Figure3D Curcumin FEV1% analysis.png]

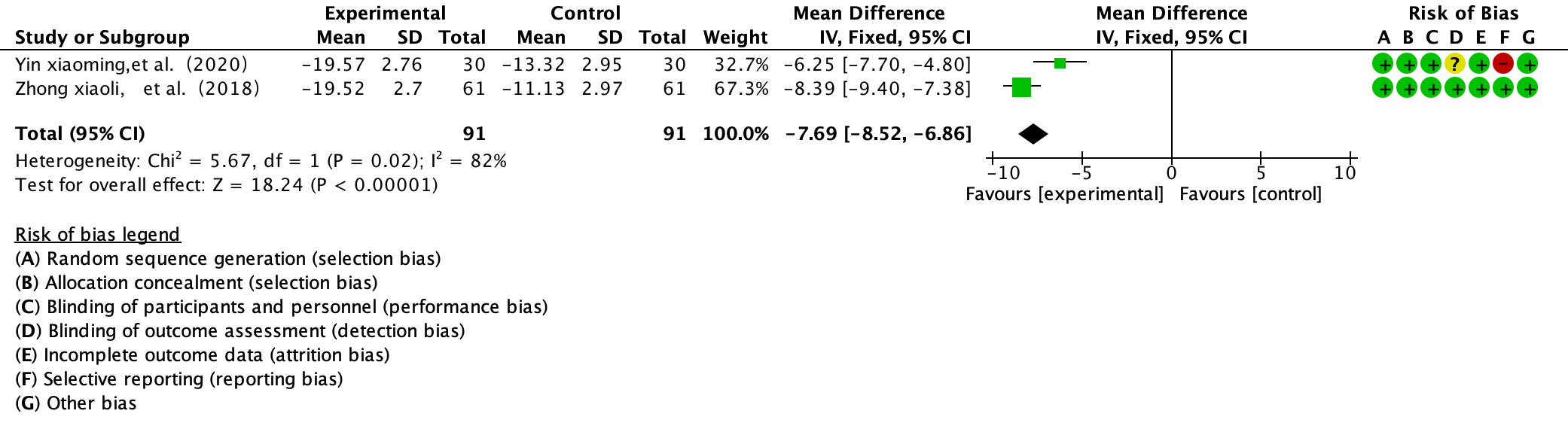

Supplement: Supplementary file 1 [file DataSheet1.zip › 3.31Efficacy and safety of dietary polyphenol supplements for COPD a systematic review and meta-analysis/Figure5E Analysis of salidroside CAT.png]

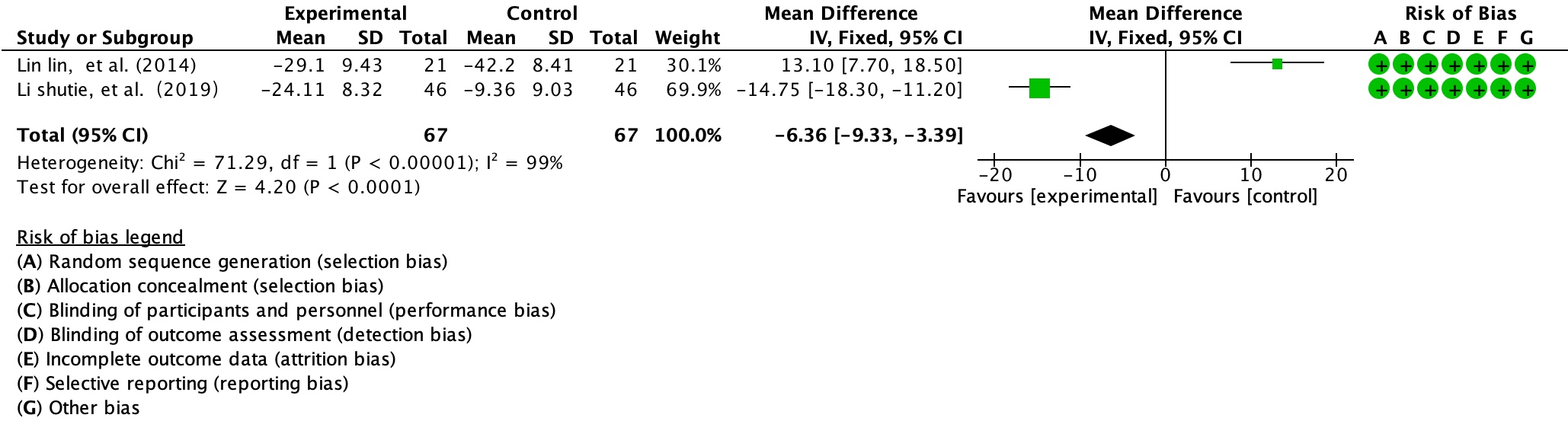

Supplement: Supplementary file 1 [file DataSheet1.zip › 3.31Efficacy and safety of dietary polyphenol supplements for COPD a systematic review and meta-analysis/Figure5F Analysis of salidroside TNF-α.png]

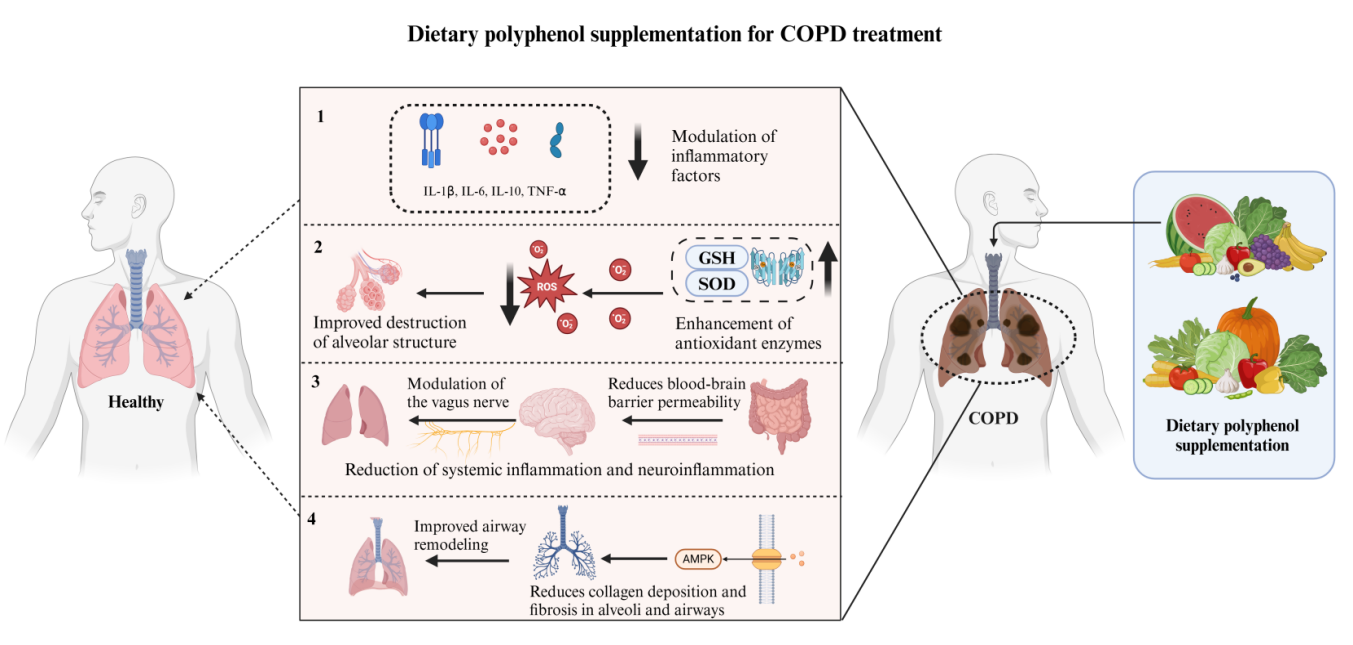

Supplement: Supplementary file 1 [file DataSheet1.zip › 3.31Efficacy and safety of dietary polyphenol supplements for COPD a systematic review and meta-analysis/Figure8 Dietary polyphenol supplementation for COPD treatment.png]
